# Structure-guided engineering of a receptor-agonist pair for inducible activation of the ABA adaptive response to drought

**DOI:** 10.1126/sciadv.ade9948

**Published:** 2023-03-10

**Authors:** Jorge Lozano-Juste, Lourdes Infantes, Irene Garcia-Maquilon, Rafael Ruiz-Partida, Ebe Merilo, Juan Luis Benavente, Adrian Velazquez-Campoy, Alberto Coego, Mar Bono, Javier Forment, Begoña Pampín, Paolo Destito, Adrián Monteiro, Ramón Rodríguez, Jacobo Cruces, Pedro L. Rodriguez, Armando Albert

**Affiliations:** ^1^ Instituto de Biología Molecular y Celular de Plantas, Consejo Superior de Investigaciones Científicas, Universidad Politécnica de Valencia, 46022 Valencia, Spain.; ^2^ Instituto de Química Física Rocasolano, Consejo Superior de Investigaciones Científicas, 28006 Madrid, Spain.; ^3^ Institute of Technology, University of Tartu, Nooruse 1, 50411 Tartu, Estonia.; ^4^ Institute of Biocomputation and Physics of Complex Systems (BIFI), Joint Units GBsC-CSIC-BIFI and ICVV-CSIC-BIFI, Universidad de Zaragoza, Mariano Esquillor s/n, 50018 Zaragoza, Spain.; ^5^ Departamento de Bioquímica y Biología Molecular y Celular, Universidad de Zaragoza, Pedro Cerbuna 12, 50009 Zaragoza, Spain.; ^6^ Instituto de Investigación Sanitaria Aragón (IIS Aragón), Avenida de San Juan Bosco 13, 50009 Zaragoza, Spain.; ^7^ Centro de Investigación Biomédica en Red en el Área Temática de Enfermedades Hepáticas y Digestivas (CIBERehd), Avenida de Monforte de Lemos, 3-5, 28029 Madrid, Spain.; ^8^ GalChimia S.A., Parque Empresarial de Touro, Parcelas 26-27, Fonte Díaz, 15822 Touro, A Coruña, Spain.

## Abstract

Strategies to activate abscisic acid (ABA) receptors and boost ABA signaling by small molecules that act as ABA receptor agonists are promising biotechnological tools to enhance plant drought tolerance. Protein structures of crop ABA receptors might require modifications to improve recognition of chemical ligands, which in turn can be optimized by structural information. Through structure-based targeted design, we have combined chemical and genetic approaches to generate an ABA receptor agonist molecule (iSB09) and engineer a CsPYL1 ABA receptor, named CsPYL1^5m^, which efficiently binds iSB09. This optimized receptor-agonist pair leads to activation of ABA signaling and marked drought tolerance. No constitutive activation of ABA signaling and hence growth penalty was observed in transformed *Arabidopsis thaliana* plants. Therefore, conditional and efficient activation of ABA signaling was achieved through a chemical-genetic orthogonal approach based on iterative cycles of ligand and receptor optimization driven by the structure of ternary receptor-ligand-phosphatase complexes.

## Teaser

Structure-guided design of a receptor-agonist pair enhances inducible activation of plant response to drought by chemical ligands.

## INTRODUCTION

Drought is a major limitation for crop productivity. Global warming and climate change exacerbate the effect of ordinary seasonal weather variations and atmospheric phenomena that limit freshwater availability, which constitutes a major threat in crop production. Plant transpiration through stomata is the major source of water loss during gas exchange for photosynthesis (*1*, *2*). Under drought stress, the phytohormone abscisic acid (ABA) controls stomatal aperture and modulates plant transpiration as well as water uptake by roots (*3*–*5*). Consequently, it has been shown that modulation of ABA response constitutes an opportunity to improve the water use efficiency (WUE) of crop plants (*6*).

Enhanced ABA levels elicited in response to drought are perceived by the PYR/PYL/RCAR family of ABA receptors and the clade A subfamily of protein phosphatases type 2C (PP2Cs), which act as necessary ABA co-receptors in ternary complexes (*7*). This leads to PP2C inhibition and concomitant activation of three ABA-activated Snf1-related protein kinases (SnRK2s) (*8*–*13*). ABA-activated SnRK2s phosphorylate ABF/AREB transcription factors and the chromatin-remodeler adenosine triphosphatase (ATPase) BRAHMA for activation of ABA transcriptional response (*14*, *15*). In plasma membrane, inhibition of K+ influx and activation of K+ efflux are achieved by phosphorylation of different K+ transporters, which together with activation of R- and S-type anion channels and aquaporins lead to loss of turgor in guard cells and stomatal closure (*16*–*19*).

The available structural information on ABA receptors provides a detailed mechanism for ABA sensing. ABA receptors are distributed into three families, i.e., subfamily III includes dimeric receptors, whereas subfamilies I and II include monomeric receptors (*20*, *21*). In the resting state, PYLR/PYL/RCAR receptors display an open ABA binding cavity flanked by two highly conserved loops, named as gate/CL2/β3-β4 loop and latch/CL3/β5-β6 loop (*10*, *22*–*25*). ABA-induced conformational rearrangements are required for dissociation of dimeric receptors and activation of monomeric receptors. Specifically, both gate and latch loops define a surface that enables the receptor to dock into the PP2C active site (*20*, *22*–*27*). The formation of receptor-ABA-phosphatase complexes causes the dissociation of different PP2C-SnRK2 complexes and abolishes PP2C-mediated inhibition of the SnRK2s, triggering ABA response (*11*, *13*, *22*, *23*, *28*–*30*). In addition, RAF-like mitogen-activated protein kinase kinase kinases (MAPKKKs) are required to reactivate SnRK2s that have been previously dephosphorylated by PP2Cs (*31*–*34*).

The detailed knowledge of this pathway has been harnessed for the development of genetic and chemical strategies to cope with drought stress, for example, the generation of agrochemical compounds mimicking ABA action as well as genetic approaches aimed to constitutively activate ABA-mediated plant response by overexpressing ABA receptors or inactivating clade A PP2C repressors (*6*, *35*–*37*). Thus, a genetic approach to reduce plant transpiration includes the overexpression of ABA receptors (*6*, *26*, *38*–*41*) or the reduced expression of the PP2C co-receptors (*6*, *35*, *42*). In addition, there is an emerging field for the development of chemical compounds that act as ABA receptor agonists (*37*, *43*–*48*) or antagonists (*49*–*51*) to modulate ABA signaling dynamically and exogenously. The chemical approach might specifically target those ABA receptors involved in regulation of plant transpiration, root water uptake, and hydrotropism (*5*, *6*). However, an additional difficulty in this field arises from the multigenic nature of PYR/PYL/RCAR ABA receptors, which show subtle sequence and structural differences (*52*). These variations usually led to a limited range of agonist activity over ABA receptors. For instance, quinabactin (QB), also known as AM1, preferentially activates ABA dimeric receptors (*44*, *45*), whereas pyrabactin is a potent agonist for PYR1 but has antagonist effect on PYL2 (*53*). The recent development of opabactin as an ABA receptor panagonist with good activity in wheat represents a step forward, although more information on its activity in other crops is needed (*36*).

The experimental identification of ABA-mimicking molecules often requires the use of expensive chemical libraries and high-throughput screening equipment. Virtual screening offers an alternative that enables in silico screening of large collections of chemical compounds. However, although chemoinformatic approaches have been improved during the last years, docking-based screening often requires extensive chemical optimization of the initial hits to obtain a lead compound. This is achieved by iterative cycles of chemical optimization followed by structural and biochemical validation. However, it is also possible to engineer the protein target to increase the affinity for the ligand and express the optimized target in plants. Thus, chemical and genetic modifications can be introduced in the optimization cycle to obtain a highly efficient receptor-agonist pair. A combination of genetic and chemical approaches may be beneficial, as the application of an agonist specific for a certain ABA receptor (in a plant overexpressing it) ensures a specific and timely ABA response (*54*, *55*). PYR1-derived receptors have been successfully designed to interact with cannabinoids and subsequently inhibit PP2C phosphatases (*56*).

Using a combination of virtual screening and efficient filtering techniques (*57*) followed by validation through PP2C inhibition assays, we were able to identify sulfobactin (SB), a sulfonamide-based molecule with agonist activity against *Arabidopsis thaliana* (arabidopsis) PYL5 and PYL10, although in vivo activity requires overexpression of its targets. Using a structure-guided approach, we have engineered a *Citrus sinensis* PYL1 (CsPYL1) variant, named CsPYL1^5m^, which shows enhanced affinity for SB. Moreover, the elucidation of the CsPYL1^5m^-SB-HAB1 structure enabled the optimization of SB. The improved SB derivatives iSB07 and iSB09 developed in this work show enhanced potency on CsPYL1^5m^ and are also active in wild-type (WT) plants. Application of low dosage of iSB09 to CsPYL1^5m^-overexpressing plants has a strong antitranspirant effect and enhances drought tolerance. Moreover, a strong ABA-like transcriptional response is achieved, which is triggered only by ligand application. Thus, using structural, chemical, genetic, and synthetic approaches, we have been able to develop a biotechnological tool with the potential to optimize crop WUE.

## RESULTS

### Identification of SB as a selective ABA receptor agonist

**Fig. 1.**
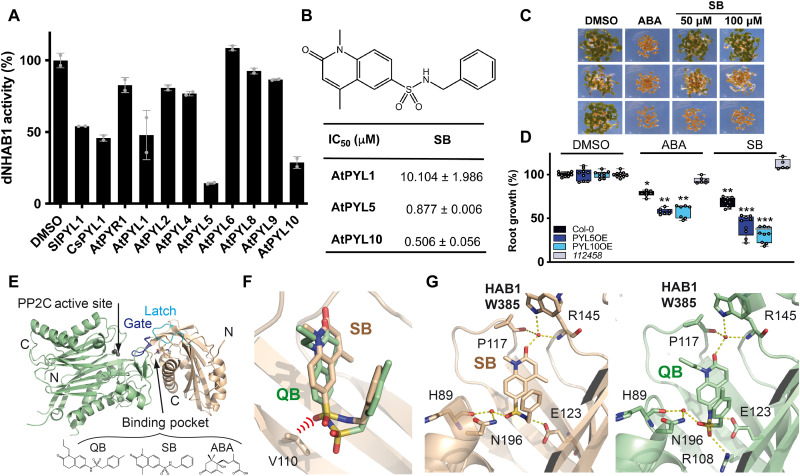
Identification of SB as a selective ABA receptor agonist and structural analysis of the CsPYL1-SB-AtHAB1ΔN complex. (**A**) PP2C inhibition assay in the presence of 100 μM SB and the indicated ABA receptors. Values represent means ± SD of two assays. (**B**) Chemical structure of SB and half-maximal inhibitory concentrations (IC_50_) values for ABA- or SB-dependent inhibition of ΔNHAB1 by AtPYL1, AtPYL5, and AtPYL10. (**C**) Inhibition of seedling establishment by 1 μM ABA and 50 to 100 μM SB in WT Col-0 and either AtPYL5- or AtPYL10-overexpressing lines. Pictures were taken at day 4. (**D**) Quantification of ABA- or SB-mediated inhibition of root growth in the indicated genotypes. Seedlings were grown in plates supplemented with 0.5% dimethyl sulfoxide (DMSO) (mock), 50 μM SB, or 10 μM ABA. Values are means ± SD of three independent experiments (*n* = 13 to 16). Asterisks indicate statistical significance (*P < 0.05, **P < 0.01, and ***P < 0.001) in Student’s *t* test compared to its corresponding DMSO-treated line. (**E**) Ribbon representation of the overall CsPYL1-SB-AtHAB1ΔN ternary complex. Ligand (SB, QB, or ABA) binding in the ternary complex occurs in the closed conformation of the receptor that fits into the active site of the HAB1 phosphatase. (**F**) Superimposition of SB and QB in the ligand-binding site of CsPYL1. The steric hinderance between the oxygen of SB’s SO_2_ group and Val^110^ of CsPYL1 is represented as red arcs. (**G**) Detailed section of the SB (left) and QB (right) binding sites showing hydrogen bonding pattern of interactions between the ligand and CsPYL1:HAB1 complex.

We performed a virtual screening to identify potential ABA agonists from a subset of molecules of the ZINC database (https://zinc.docking.org/) using the structure of the CsPYL1-ABA-HAB1 complex (*7*) as target (fig. S1). The compounds of the chemical library were selected to be large enough as to fit into the ABA binding pocket of CsPYL1 and to harbor a number of hydrogen bonding acceptor and donor atoms to match the characteristics of the pocket in the ternary complex. After docking, we filtered the hits according to their potential agonist activity using descriptors that quantify the presence of acceptor groups in the vicinity of the ABA’s ketone and carboxylate groups and hydrophobic atoms in the region comprised between these two groups. After visual inspection of the best-fitting molecules, five compounds were purchased and tested by their ability to inhibit the arabidopsis PP2C HAB1 in the presence of *C. sinensis* (sweet orange) CsPYL1 (fig. S1). Further work was focused on *N*-benzyl-1,4-dimethyl-2-oxo-1,2-dihydroquinoline-6-sulfonamide because it showed in vitro activity (Fig. 1, A and B). This compound was named as “sulfobactin” (SB) to highlight the arrangement of the sulfonamide moiety (see below). SB does not share chemical similarity with ABA, but it does with other sulfonamides, e.g., QB and pyrabactin, that are selective ABA receptor agonists (*8*, *44*, *45*). These molecules consist of a sulfonamide-based three-atom linker joining two aromatic rings. The position of the sulfonamide moiety in the SB linker (-SO_2_-NH-CH_2_-) differs from QB, where the sulfonamide linkage is reversed with respect to SB (-NH-SO_2_-CH_2_-) (Fig. 1, B and E).

We investigated further the in vitro activity of SB with different ABA receptors, including *Solanum lycopersicum* (tomato) SlPYL1 and representative *A. thaliana* ABA receptors (Fig. 1, A and B, and fig. S1) (*7*). ABA receptors are able to inhibit PP2Cs from different plant species, and we chose HAB1 because successful procedures for its crystallization have been previously reported (*7*, *27*, *30*). Regarding arabidopsis receptors, we included representative members of the three receptor subfamilies, such as PYR1, PYL1, PYL2, PYL4, PYL5, PYL6, PYL8, PYL9, and PYL10 (*58*). Using 100 μM SB, both PYL5 and PYL10 were able to inhibit the HAB1 phosphatase activity by more than 75%, whereas AtPYL1, CsPYL1, and SlPYL1 (PYL1 family) induced around 50% reduction of phosphatase activity. To quantitatively characterize SB activity with respect to ABA, we determined the half maximal inhibitory concentrations (IC_50_) of SB on HAB1 activity with either AtPYL1, AtPYL5, or AtPYL10. The IC_50_ values of SB using PYL1 and PYL5 were circa 30-fold higher than those calculated for ABA. In contrast, the IC_50_ of SB with AtPYL10 is only fourfold higher than the IC_50_ of ABA (Fig. 1B) (*45*). Whereas SB activates efficiently AtPYL10 and only moderately AtPYL1, QB activates AtPYL1 but not AtPYL10 (*45*). AtPYL5 displays similar sensitivity to SB and QB (*44*, *45*). Next, we tested the in vivo activity of SB on WT Col-0 and either PYL5- or PYL10-overexpressing lines. SB reduced the seedling establishment of both PYL5- and PYL10-overexpressing lines but was not active in WT seeds even at 100 μM (Fig. 1C). These results suggest that SB is a weak agonist that is more active in vivo when its targets are overexpressed. This effect was also observed in root growth assays because SB reduced root growth by 40% in PYL5- and PYL10-overexpressing lines but only 20% in WT seedlings (Fig. 1D). In contrast, the sextuple *112458* mutant was resistant to SB, which indicates that SB effect on root growth is dependent on ABA receptors (Fig. 1D) (*58*). In summary, SB is a selective ABA receptor agonist that, in contrast to previously described sulfonamide-based ABA agonists, targets PYL10 (*46*).

### Structural analysis of the SB binding pocket in CsPYL1

As a first step to improve binding of SB to CsPYL1, we determined and compared the crystal structure of CsPYL1-SB-HAB1 and CsPYL1-QB-HAB1 complexes (Fig. 1, E to G, and table S1). The gate and latch loops of CsPYL1 display a closed conformation that fits into the active site of the HAB1 phosphatase (Fig. 1E). Both ternary complexes yield isomorphous crystals whose molecular structure was almost identical to the previously reported CsPYL1-ABA-HAB1 complex (*7*). SB and QB display a “U”-shaped conformation into the ligand-binding pocket of the receptor (Fig. 1F). However, superimposition of the structures of the CsPYL1-SB-HAB1 and CsPYL1-QB-HAB1 ternary complexes reveals a shallower insertion of SB in the ABA binding pocket with respect to that observed for QB. This may be explained by the different orientation in SB and QB of the SO_2_ group, which contains one oxygen atom that shows similar position in both compounds and interacts through a water bridge with both Asn^196^ and the backbone carbonyl of His^89^, whereas the other oxygen is located in different position in SB and QB. Thus, in the QB complex, this latter oxygen forms a hydrogen bond with Arg^108^, whereas in the SB complex it produces steric hindrance with the Val^110^ side chain of CsPYL1 (Fig. 1, F and G). This difference in SO_2_ coordination may explain the reduced activity of SB against dimeric ABA receptors, compared to QB (Fig. 1B) (*45*). Despite this major difference, QB and SB maintain otherwise a similar network of hydrogen bond interactions in the ABA binding pocket. These include the hydrogen bonds linking the sulfonamide NH to Glu^123^ side chain, the SO_2_ group to a conserved water molecule described above, and the hydrogen bond network forming the “Trp lock” (*22*). This is depending on a water molecule that is hydrogen-bonded to the carbonyl oxygen of SB or QB, the backbone amine of Arg^145^ at the latch and the carbonyl of Pro^117^ at the gate, and the side chain of Trp^385^ from HAB1 (Fig. 1G).

### Engineering a synthetic CsPYL1^5m^ receptor with enhanced sensitivity to SB

**Fig. 2.**
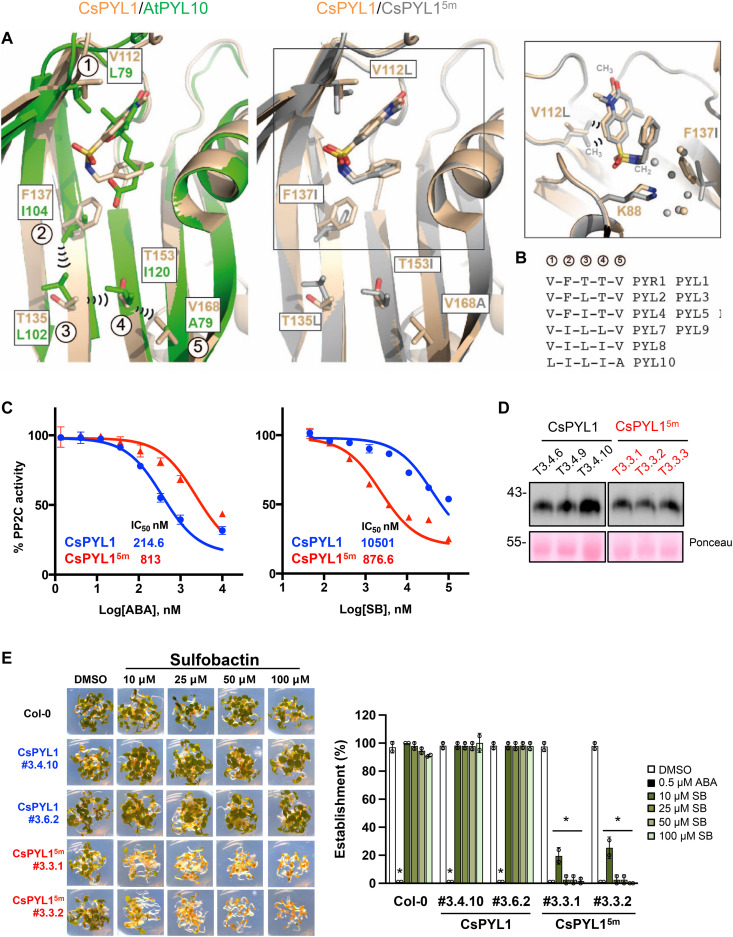
Structure-guided design of a synthetic CsPYL1^5m^ receptor that shows enhanced sensitivity to SB. (**A**) Superimposition of the ligand-binding pocket of CsPYL1-SB-AtHAB1ΔN (wheat) and either PYL10-ABA (green) (left) or CsPYL1^5m^-SB-AtHAB1ΔN (gray) (middle and right). The concatenated interactions of the four PYL10 residues (labeled from 2 to 5) are indicated as black arcs. The right panel highlights the higher water network (gray spheres) and methyl contacts of Leu^112^ with SB (lacking in Val^112^ of CsPYL1) in the ligand-binding site of CsPYL1^5m^. Residues changed in CsPYL1^5m^ are labeled in gray (middle and right). (**B**) The chain of interacting residues along the β sheet in PYR1 to PYL10 receptors is labeled from 2 to 5, according to the structural detail shown in (A). Number 1 corresponds to Leu^79^ of PYL10 and the equivalent position in other receptors. (**C**) Determination of the IC_50_ (nM) for inhibition of HAB1 by ABA and SB in the presence of CsPYL1 (blue circles) or CsPYL1^5m^ (red triangles). Dose-response curves are shown in the presence of the indicated concentrations of ABA or SB. (**D**) Immunoblot analysis of protein extracts obtained from CsPYL1 and CsPYL1^5m^ lines. The epitope-tagged receptor was detected using anti-HA antibodies. Ponceau staining serves as a protein loading control. (**E**) Inhibition of seedling establishment by SB in WT Col-0 and either CsPYL1- or CsPYL1^5m^-overexpressing lines. Representative images are shown in the left panel, and quantification of the experiment is shown in the right panel. Values are means ± SD of two independent experiments (*n* = 20 each). *P < 0.05, Student’s *t* test, compared to Col-0.

Given that PYL10 is 20-fold more sensitive to SB than PYL1 members (Fig. 1B), we aimed to engineer a mutated version of CsPYL1 with enhanced sensitivity to SB by taking advantage of the CsPYL1-SB-HAB1 complex and PYL10 structural features. First, we superimposed the crystal structure of CsPYL1-SB-HAB1 with that of the AtPYL10-ABA complex [Protein Data Bank (PDB) code: 3R6P] and compared their binding pockets (Fig. 2A). This analysis revealed that the main differences are found at the opposite sides of the binding pocket; specifically, the Val^112^ of CsPYL1 is substituted by the bulkier Leu^79^ of AtPYL10. On the other hand, Phe^137^ of CsPYL1 is substituted by the smaller Ile^104^ of AtPYL10 (Fig. 2A). For a deeper analysis, we performed amino acid sequence alignment of arabidopsis ABA receptors to check which residues occupy equivalent positions to Leu^79^ and Ile^104^ in the three major subfamilies (fig. S2A). The Leu^79^ residue is a unique feature of AtPYL10 since this position is occupied by Val in all other receptors (Fig. 2B and fig. S2A). In addition, a deeper analysis of the CsPYL1 and AtPYL10 structures suggests that the Phe^137^ to Ile^104^ change, discussed above, is likely coupled with additional changes that form a chain of variable and interacting residues along the β sheet, including Thr^135^ to Leu, Thr^153^ to Ile, and Val^168^ to Ala (residues labeled as 1 to 5 from top to bottom; Fig. 2A, left). As this chain of interacting residues seems to be a characteristic of PYL10, we reasoned that these changes might explain the enhanced activity of SB against PYL10 compared to PYL1 receptor.

**Fig. 3.**
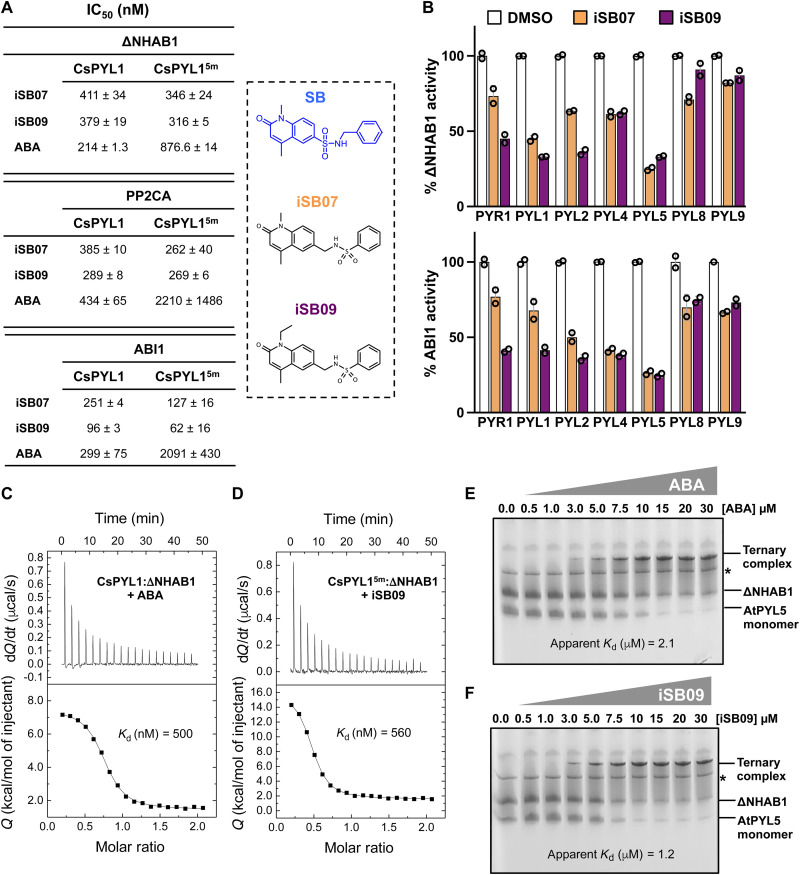
iSB07 and iSBi09 are SB derivatives that show improved agonist activity. (**A**) Chemical structure of iSB07 and iSB09 showing the swap of the SO_2_ group and CH_2_ of the benzyl group with respect to SB structure. The table shows the IC_50_ (nM) for inhibition of AtHAB1ΔN, PP2CA, and ABI1 by iSB07 and iSB09 in the presence of CsPYL1 or CsPYL1^5m^ using pNPP as substrate. (**B**) PP2C inhibition assay in the presence of 1 μM iSB07 or iSB09 and the indicated arabidopsis ABA receptors. For AtHAB1ΔN, pNPP was used as a substrate; for ABI1, phosphopeptide was used as a substrate. Values represent means ± SD of two assays. (**C** and **D**) The binding of ABA to CsPYL1 in the presence of ΔNHAB1 shows similar affinity to the binding of iSB09 to CsPYL1^5m^. ITC data were obtained by repeated injections of ABA or iSB09 into a 1:1 mixture of receptor:ΔNHAB1. (**E** and **F**) Native red electrophoresis (NRE) analysis of ligand-induced ternary complexes. Dose-response NRE analysis of ABA-induced (E) or iSB09-induced (F) AtPYL5-ligand-ΔNHAB1 complex. The fraction of ligand bound in the ternary complex was represented against free ABA or free iSB09 concentration to calculate apparent *K*_d_.

To improve the binding of the SB ligand into CsPYL1, we translated the aforementioned features of AtPYL10 into the ABA binding pocket of CsPYL1 by engineering a synthetic CsPYL1 receptor including five substitutions, Val^112^Leu, Phe^137^Ile, Thr^135^Leu, Thr^153^Ile, and Val^168^Ala (abbreviated as CsPYL1^5m^) (Fig. 2A, middle), and performed biochemical assays to analyze the sensitivity of CsPYL1^5m^ to SB and ABA (Fig. 2C). We tested that the introduction of these five mutations into CsPYL1^5m^ does not alter the dimeric state of the receptor (fig. S2B). We observed increased sensitivity to SB of CsPYL1^5m^ with respect to the WT as the IC_50_ of SB was 12-fold lower for the synthetic receptor (Fig. 2C). In contrast, a fourfold increase in the IC_50_ of ABA was determined in the synthetic receptor compared to its WT (Fig. 2C). To further explain these results, we solved the crystal structures of CsPYL1^5m^-SB-HAB1 and CsPYL1^5m^-ABA-HAB1 complexes and compared them with those obtained previously for WT CsPYL1 (Fig. 2A, middle, and fig. S3). The comparison of CsPYL1 and CsPYL^5m^ binding pockets reveals that CsPYL1^5m^ displays a closer interaction with SB at mutated Leu^112^, whose methyl groups show favorable contacts with the SB’s aromatic bicyclic ring (Fig. 2A, middle and right) (*59*). In addition, an enlargement of the binding pocket at Ile^137^ is observed (Fig. 2A, right). This free space is filled with water molecules that interact with the polar side chain of Lys^88^, which is otherwise buried in a more hydrophobic environment in the WT receptor bound to SB (Fig. 2A, right). Thus, Lys^88^ of CsPYL1^5m^ is more amenable to polar interactions in the pocket of CsPYL1^5m^ bound to SB. Together, these structural insights may explain the enhanced sensitivity of CsPYL1^5m^ to SB. The synthetic CsPYL1^5m^ receptor displays a similar chain of stabilizing interactions as AtPYL10 along the five mutated residues (Fig. 2A, left and middle). However, it is difficult to foresee the precise contribution of a distant mutation, such as Val^168^Ala, to reshape the ligand-binding pocket to better accommodate SB. Nevertheless, distant mutations from the binding pocket of AtPYR1 were required to bind mandipropamid (MD) and trigger activation of an engineered receptor (*54*). The comparison of the ternary complexes of CsPYL1 or CsPYL1^5m^ with ABA and HAB1 reveals that there is a reduction of the water-mediated hydrogen bonds that bind ABA to the receptor in the vicinity of the carboxylate group, which may explain the diminished ABA sensitivity of CsPYL1^5m^ with respect to the WT (fig. S3, A to C; see below Fig. 3A). Both HAB1 and ABI1 were less inhibited by ABA in the presence of CsPYL1^5m^ than with CsPYL1 (fig. S3, B and C; see below Fig. 3A reporting IC_50_ of ABA for three clade A PP2Cs).

Next, we generated arabidopsis transgenic plants expressing CsPYL1^5m^ driven by the 35*S* promoter and, as a control, we also generated transgenic plants expressing the WT version, i.e., *35S:CsPYL1* (Fig. 2D). We selected T3 plants expressing similar levels of CsPYL1 and CsPYL1^5m^ and analyzed their sensitivity to ABA and SB (Fig. 2, D and E). Both *35S:CsPYL1* and *35S:CsPYL1^5m^* plants showed similar sensitivity to ABA-mediated inhibition of seedling establishment (Fig. 2E, right). In accordance with the in vitro IC_50_ of SB for CsPYL1 and CsPYL1^5m^, we used higher concentrations of this compound than ABA in the seedling establishment assay (Fig. 2E). *35S:CsPYL1^5m^* lines were sensitive to 10 μM SB, whereas 35*S*:CsPYL1 lines were not affected even by 100 μM SB (Fig. 2E). These results indicate that structure-guided modifications of CsPYL1^5m^ were effective in vivo and suggest increased capability of the mutated receptor to bind SB. However, the dosage of SB required to inhibit seedling establishment of CsPYL1^5m^ lines was still 20-fold higher than ABA.

### Development of iSB derivatives with enhanced activity as ABA receptor agonists by swapping of SO_2_ and CH_2_ in SB’s sulfonamide linker

The wealth of crystallographic information on ABA receptors in complex with agonist and antagonist molecules has revealed key structural features of the ligand-receptor coordination that enable ligand improvement. To optimize the SB scaffold for enhanced activation of ABA receptors, we first explored substitutions in the dihydroquinoline of SB to fill the 3′ tunnel of ABA receptors and synthesized different SB derivatives (fig. S4A) (*46*). The 3′ tunnel is a small solvent-exposed space adjacent to ABA’s 3′-CH that normally interacts with ABA’s 7′ methyl group (*46*, *50*). The 3′ tunnel is formed by five highly conserved hydrophobic residues (in PYR1: Phe^61^, Leu^87^, Pro^88^, Phe^159^, and Val^163^) and can accept certain alkyl substituents of ABA agonists to form hydrophobic contacts and increase agonist potency (*45*, *46*, *50*). Introduction of 1-ethyl instead of the 1-methyl group in the dihydroquinoline, i.e., compound SB-01, led to enhanced inhibitory activity of the agonist with both CsPYL1 and CsPYL1^5m^ (fig. S4A). In the case of CsPYL1^5m^, we found that SB-01 showed enhanced inhibition of seedling establishment compared to SB in CsPYL1^5m^ lines (fig. S4B). Introduction of a bulkier hydrophobic substituent as the cyclopropyl group in SB-02 did not improve the in vitro capability of the agonist to inhibit HAB1 activity compared to SB-01 (fig. S4A). On the other hand, we also introduced changes in the SB’s benzyl ring, i.e., SB-03 to SB-06. SB-05 and SB-06 showed less effect than SB and were not investigated further (fig. S4A). Introduction of the methyl substituent in SB-03 led to some improvement compared to SB, but no improvement compared to SB-01 (fig. S4, A and B).

Recently, opabactin has been described as a powerful ABA agonist and a key feature for its enhanced interaction with ABA receptors in the presence of a carboxylate group that interacts with a conserved Lys residue in the ABA binding pocket (*36*). Both CsPYL1-SB-HAB1 and CsPYL1^5m^-SB-HAB1 complexes lack such interaction of the agonist with the conserved Lys^88^ residue of the receptor; rather, the structures show that the hydrophobic CH_2_ of the SB linker faces a water-filled cavity harboring the side-chain NH_3_^+^ of Lys^88^ (Fig. 2A, middle and right). This water-filled cavity in CsPYL1^5m^ might enable the design of SB derivatives with increased potency. We reasoned that swapping the positions of the SO_2_ and CH_2_ moieties in the SB linker might contribute to fill this cavity by promoting the formation of a hydrogen bond between the SO_2_ moiety of the agonist and the Lys^88^ side chain, increasing ligand activity. We, therefore, synthesized two new compounds incorporating the above swap, iSB07 and iSB09 (“inverted SB”) (see Fig. 3A and the Supplementary Materials). Compared to iSB07, iSB09 contains a larger alkyl group (ethyl versus methyl) at the dihydroquinoline ring that might increase ligand activity through interaction with the 3′ tunnel as described above for SB-01 (see below Fig. 3C). The HAB1 IC_50_ values of SB, iSB07, and iSB09 combined with CsPYL1^5m^ were 876, 346, and 316 nM, respectively, which indicates that both iSB07 and iSB09 are improved versions of SB (compare Figs. 2C and 3A). This improvement was also evident when HAB1 IC_50_ of iSB07 and iSB09 was obtained in combination with CsPYL1 (Figs. 2C and 3A), compared to SB. We extended the iSB07/iSB09-dependent PP2C inhibition assay to ABI1 and PP2CA, which are phosphatases very relevant for ABA signaling (*42*). Both ABI1 and PP2CA were even more sensitive to iSB09-mediated CsPYL1^5m^-dependent inhibition, particularly ABI1 was fivefold more sensitive than HAB1 (Fig. 3A). Last, we also tested the action of iSB07 and iSB09 with seven ABA receptors that are biologically relevant in arabidopsis (Fig. 3B). iSB09 was more active toward dimeric receptors than iSB07, and using 1 μM iSB09, 60 to 70% inhibition of HAB1 was achieved when the dimeric PYR1, PYL1, and PYL2 as well as monomeric PYL5 were assayed (Fig. 3B). Comparison of these results with those obtained for 100 μM SB (Fig. 1A) also reveals a marked improvement of the agonist potency in iSB compounds, being the ethyl group of iSB09 an additional improvement compared to the methyl group of iSB07 (Fig. 3B).

We also conducted isothermal titration calorimetry (ITC) experiments comparing ABA-CsPYL1-HAB1 and iSB09-CsPYL1^5m^-HAB1 binding reactions (Fig. 3, C and D). These experiments show a similar *K*_d_ (dissociation constant) for ABA and iSB09 binding in these ternary complexes, which suggests that the combination iSB09-CsPYL1^5m^-HAB1 matches the affinity of ABA binding in the ABA-CsPYL1-HAB1 complex. We also performed additional experiments to examine the formation of ligand-induced ternary complexes by using native red electrophoresis (NRE) (Fig. 3, E and F). In this case, we examined ABA-AtPYL5-HAB1 and iSB09-AtPYL5-HAB1 binding reactions by NRE. A dose-response NRE analysis followed by quantification of the ternary complex formed versus free ABA or iSB09 concentration also revealed a similar apparent *K*_d_ for both ligands. Last, comparison of ABA and iSB09 effect in ABI1 inhibition assays showed that IC_50_ values for AtPYR1 or AtPYL1 were similar for both ligands (fig. S3D). Therefore, we conclude that the swapping of SO_2_ and CH_2_ in SB’s sulfonamide linker leads to iSB derivatives that match ABA efficiency for the formation of certain ternary ligand-receptor-phosphatase complexes.

### Structural insights into iSB-receptor-phosphatase complexes

**Fig. 4.**
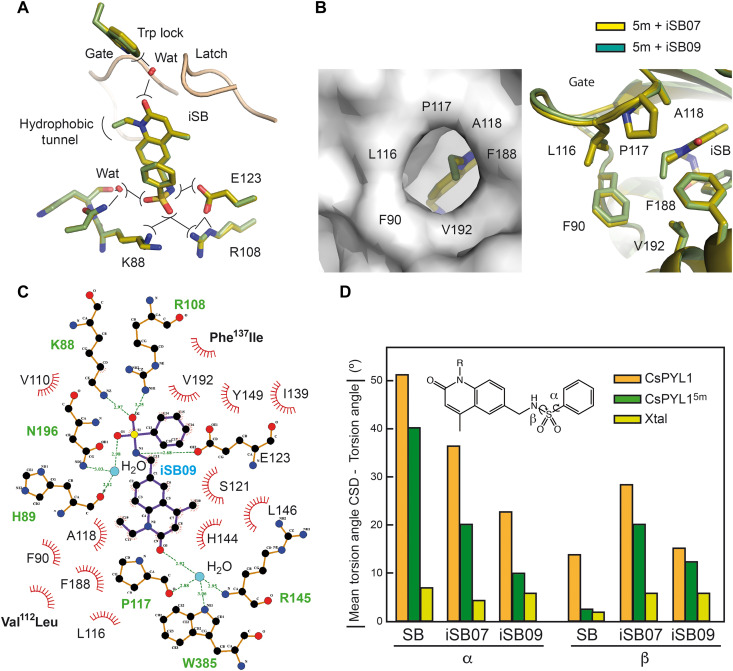
Structural insights into iSB receptor–phosphatase complexes. (**A** and **B**) Superimposition of the ligand-binding pocket in CsPYL1-iSB07-AtHAB1ΔN, CsPYL1^5m^-iSB07-AtHAB1ΔN, CsPYL1-iSB09-AtHAB1ΔN, and CsPYL1^5m^-iSB09-AtHAB1ΔN complexes. (A) Interactions at the Trp lock (top) and the hydrogen bond network in the opposite part of the ligand (bottom). (B) Hydrophobic tunnel of the receptors and interactions of the alkyl group close to the carbonyl oxygen. (**C**) Two-dimensional schematic representation of the iSB09 interactions in the ligand-binding pocket of the CsPYL1^5m^-ligand-AtHAB1ΔN ternary complex. (**D**) SB, iSB07, and iSB09 in complex with CsPYL1^5m^ relax to a more stable conformation than that observed in complex with CsPYL1. The α and β torsion angles along the sulfonamide linker of these ligands served to characterize their conformation. The bar diagram represents the differences between the mean value of torsion angles α and β observed for identical fragments recorded at CSD and those values in the pure compounds (Xtal) and in complex with CsPYL1 and CsPYL1^5m^.

To gain insight into the structural basis of iSB agonist activity, we solved the crystal structures of CsPYL1-iSB07-HAB1, CsPYL1-iSB09-HAB1, CsPYL1^5m^-iSB07-HAB1, and CsPYL1^5m^-iSB09-HAB1 (Fig. 4, A to C, and table S1). Consistent with our docking predictions, both iSB07 and iSB09 complexes display the hydrogen bond network to conform the Trp lock and the compounds show a hydrogen bond between the oxygen of the SO_2_ moiety and side-chain NH_3_^+^ of Lys^88^ and, unexpectedly, an additional one to the guanidinium moiety of Arg^108^ side chain (Fig. 4, A and C). Thus, the unique chemical features of the iSB07 and iSB09 sulfonamide linker are central for the formation of these additional H-bonds and likely explain the enhanced activity of these compounds with respect to that observed for SB, as SB lacks these interactions (compare Figs. 1G and 4A and fig. S5A). In addition, iSB09 structures reveal that iSB09’s ethyl group, compared to iSB07’s methyl group, increases the number of hydrophobic contacts at the 3′ tunnel without distorting the conformation of the gate (Fig. 4, B and C). In addition, there are several favorable rearrangements in the conformation of the iSB07, iSB09, and SB molecules in the ternary complex with CsPYL1^5m^ compared to CsPYL1. Specifically, the structure of the sulfonamide linker displays a more likely and relaxed conformation (in terms of the torsion angles) for agonists in the ligand-binding pocket of CsPYL1^5m^ than in CsPYL1. Thus, the molecular geometries of the ligands in the ternary complexes with CsPYL1^5m^ are more relaxed, showing torsion values closer to the maximum values observed for the ligands in the unbound form, available from the Cambridge Structural Database (CSD) (Fig. 4D and fig. S5) (*60*, *61*). Together, the structural data suggest that the differences observed for iSB09, iSB07, and SB potency as agonists may be a consequence of a number of adjustments in the conformation of the ligand as well as interactions in the binding pocket of the receptors, which include the formation of additional hydrogen bonds and favorable hydrophobic contacts for iSB compounds.

### Enhancement of drought tolerance by application of iSB09 to 35*S*:CsPYL1^5m^ plants

**Fig. 5.**
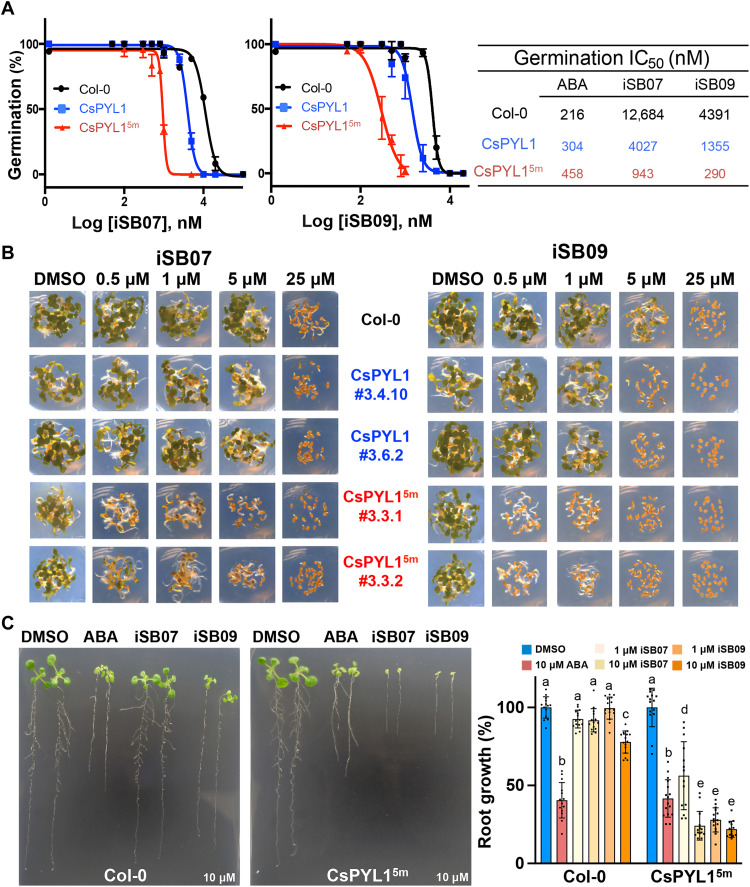
The iSB07 and iSB09 compounds show enhanced agonist potency in vivo combined with the synthetic CsPYL1^5m^ receptor. (**A**) Determination of the IC_50_ for inhibition of seed germination by iSB07 and iSBi09 in WT Col-0 or in lines expressing CsPYL1 or CsPYL1^5m^ receptors. Values represent means ± SD of three assays. (**B**) Inhibition of seedling establishment by iSB07 and iSB09 in WT Col-0 or in lines expressing CsPYL1 or CsPYL1^5m^ receptors. Pictures were taken at day 4. The experiment was repeated at least twice with similar results. (**C**) ABA- or iSB-mediated inhibition of root growth in the indicated genotypes. Representative images of the different treatments and genotypes are shown along with the quantification of root growth (right). Values are means ± SD (*n* = 13 to 16) of relative growth compared to Col-0 in control conditions (0.1% DMSO). Different letters indicate statistical significance by one-test analysis of variance (ANOVA).

The combination of genetic and chemical approaches is a powerful tool to enhance ABA signaling (*54*, *55*); therefore, we tested the application of iSB07 and iSB09 in arabidopsis transgenic plants that express either the WT CsPYL1 or the synthetic CsPYL1^5m^ receptor. We performed different assays in seeds and vegetative tissues to evaluate ABA responses after applying the compounds in these plants. Plants expressing CsPYL1^5m^ were markedly more sensitive to agonist-mediated inhibition of seed germination compared to plants expressing the WT version (Fig. 5A). Moreover, iSB09 was threefold more effective than iSB07 to inhibit seed germination in plants expressing CsPYL1^5m^ (data in Fig. 5A, right). Likewise, inhibition of seedling establishment by iSB07 and iSB09 was markedly enhanced in plants expressing CsPYL1^5m^ compared to CsPYL1 lines (Fig. 5B). Moreover, the iSB09 compound was able to inhibit seedling establishment of WT Col-0 at 5 μM, whereas this dosage was reduced to 0.5 μM in plants that express CsPYL1^5m^ (Fig. 5B). Next, we tested iSB07- and iSB09-mediated inhibition of root growth in 5-day-old seedlings of WT Col-0 and CsPYL1^5m^ lines (Fig. 5C). At 10 μM concentration, iSB07 and iSB09 did not significantly inhibit root growth of WT plants; however, a marked inhibition of CsPYL1^5m^ lines was observed. Therefore, we conclude that the combination of iSB07 or iSB09 with CsPYL1^5m^ is a powerful tool to activate ABA signaling and promote ABA responses.

**Fig. 6.**
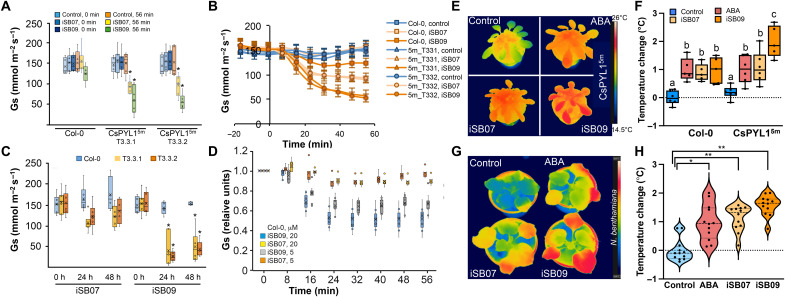
Whole-plant gas exchange and thermal imaging analysis after chemical treatment. (**A**) Stomatal conductance (Gs) values of WT Col-0 and two lines expressing CsPYL1^5m^ 56 min after spraying with 5 μM iSB07/iSB09 or 0.1% DMSO (control). Asterisk in (A) and (C) denotes significant differences with respect to the pretreatment value of stomatal conductance (repeated-measures ANOVA, Generalized Linear Model, GLM). Values show averages ± SE, *n* = 5 to 7. (**B**) Time courses of Gs after spraying with 5 μM iSB07/iSB09 or control solutions. Values show averages ± SE, *n* = 5 to 7. (**C**) Gs values of WT Col-0 and two transgenic lines before or 24 and 48 hours after spraying with 5 μM iSB07/iSB09 or control. Values show averages ± SE, *n* = 4 to 7. (**D**) Time courses of Gs in relative units for WT Col-0 after spraying with 5 or 20 μM iSB07/iSB09. Only treatment with 20 μM iSB09 led to a significant reduction of stomatal conductance (repeated-measures ANOVA, GLM). Values show averages ± SE, *n* = 5 to 7. (**E**) IR images of representative arabidopsis CsPYL1^5m^ plants 24 hours after being treated with 0.1% DMSO (control), 50 μM ABA, iSB07, or iSB09. (**F**) Quantification of the temperature difference for the experiment described in (E). The temperature of 15 different sectors corresponding to four to six leaves per plant was quantified as described in Materials and Methods. The values represent the temperature increase versus control. Statistical analysis (one-way ANOVA) was performed using GraphPad Prism 9. Different letters indicate statistical significance. At least six plants per genotype and treatment were analyzed. The experiment was repeated twice. (**G**) IR images of representative *N. benthamiana* WT plants treated with 0.1% DMSO (control), 50 μM ABA, or 100 μM iSB07/iSB09. IR images were obtained 24 hours after the treatment. (**H**) Quantification of the experiment described in (G). The values represent the temperature increase versus control.

Last, we tested the capability of iSB07 and iSB09 to regulate stomatal aperture in WT Col-0 and CsPYL1^5m^ lines by whole-plant gas exchange analysis of stomatal conductance (Gs) after treating with the compounds. Spraying with 5 μM iSB07 or iSB09 had a significant effect on Gs in the transgenic lines expressing CsPYL1^5m^ [repeated-measures analysis of variance (ANOVA), Fig. 6A; detailed time course, Fig. 6B]. The dynamic courses of Gs after spraying show that the reduction of Gs induced by iSB07 in transgenic lines was significant but lower compared to iSB09 (Fig. 6B). There were no significant differences between the lines and WT in basal pretreatment Gs (Fig. 6B). In WT, the small effect of 5 μM iSB09 was nonsignificant, but spraying with 20 μM iSB09 was effective to reduce Gs, whereas iSB07 was not effective in WT even at 20 μM (Fig. 6C). In transgenic lines, reduction of Gs by iSB09 was still significantly evident 24 and 48 hours after spraying, with no signs of recovery (Fig. 6D). Therefore, the iSB09 effect lasted at least for 48 hours. The Gs of transgenic lines was lower compared to pretreatment values 24 and 48 hours after spraying with iSB07; however, these differences were nonsignificant (Fig. 6D).

**Fig. 7.**
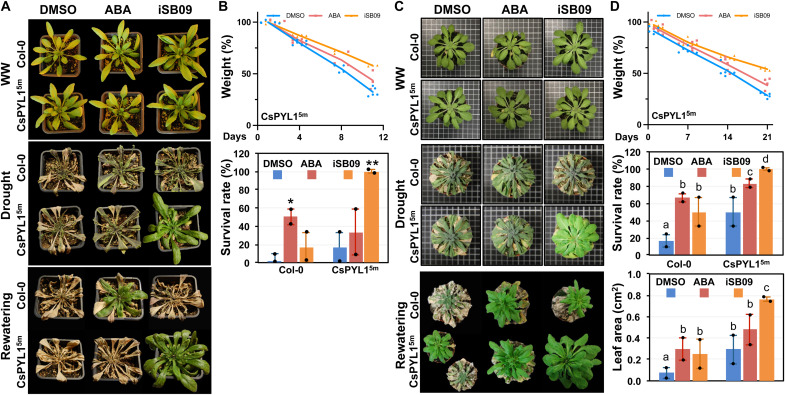
Drought resistance assays under long-day or short-day conditions. (**A**) WT Col-0 and CsPYL1^5m^ plants grown under long-day conditions were submitted to water deprivation and treated with 0.1% DMSO (mock), 50 μM ABA, or iSB09 as described in Materials and Methods. Photographs show representative plants (at least 50% of the total cases) in well-watered (WW) conditions, submitted to drought and after rewatering. (**B**) Gravimetric analysis (top) of water loss in pots containing CsPYL1^5m^ plants reveals reduced water consumption in plants treated with ABA and iSB09 compared to mock-treated plants. The percentage (%) of weight relative to day 1 is shown and reflects water remaining in soil along the drought experiment. Six plants per genotype and treatment were analyzed. The experiment was performed twice. Bottom: Survival rate of Col-0 and CsPYL1^5m^ plants 6 days after rewatering. Values indicate means ± SD. Asterisks indicate statistical significance (*P < 0.05 and **P < 0.01) in Student’s *t* test compared to their corresponding DMSO-treated genotype. (**C**) WT Col-0 and CsPYL1^5m^ plants grown under short-day conditions were submitted to water deprivation and treated with 0.1% DMSO (mock), 50 μM ABA, or iSB09 as described in Materials and Methods. (**D**) Gravimetric analysis (top) was performed as described above. Middle: Survival rate of Col-0 and CsPYL1^5m^ plants 12 days after rewatering. Bottom: Enhanced growth of leaves in CsPYL1^5m^ plants treated with iSB09 compared to mock- or ABA-treated plants. Values indicate means ± SD. Different letters indicate statistical significance by one-way ANOVA.

Thermal imaging in transgenic lines revealed increased leaf temperature upon spraying with both iSB07 and iSB09 (Fig. 6, E and F), which also occurred in nontransformed Col-0 plants (Fig. 6F). Last, thermal imaging of nontransformed *Nicotiana benthamiana* leaves 24 hours after spraying with 50 μM ABA or 100 μM iSB07 or iSB09 also revealed increased leaf temperature compared to mock-treated [dimethyl sulfoxide (DMSO)] plants (Fig. 6, G and H). Application of the iSB09 compound was the most effective to increase leaf temperature, which indicates that this compound can also promote stomatal closure in WT plants, in either *A. thaliana* or *N. benthamiana* (Fig. 6, F and H). *N. benthamiana* shows a high biomass production and transpiration, so these results suggest that spraying of iSB09 might even be effective as antitranspirant treatment in crops that show high ratios of transpiration. As a proof of concept, we tested iSB09 bioactivity in tomato at 10 μM and used thermal imaging to measure its effect as an antitranspirant (fig. S6, A and B). Quantification of the leaf temperature by infrared (IR) thermography analyses indicated that iSB09’s effect in tomato was present for up to 5 days after spraying, relative to mock- or QB-treated plants (fig. S6A). QB lacked bioactivity in tomato, in agreement with previous results reported by Vaidya *et al*. (*36*), whereas iSB09 was active and showed long-lasting effects (fig. S6A). Given that the iSB09 effect on Gs was markedly enhanced in lines expressing CsPYL1^5m^, we decided to test whether spraying of this compound over CsPYL1^5m^ plants during a drought period was effective to enhance drought resistance, reduce water consumption (increasing water retention in the soil, “water banking”), and promote survival of the plants after rewatering (Fig. 7). We devised two drought treatments, either in a plant growth chamber under short-day conditions to favor rosette development (Fig. 7, C and D) or in greenhouse under long-day conditions (Fig. 7, A and B). Figure 7A shows that iSB9 treatment markedly enhanced drought resistance of CsPYL1^5m^ plants grown under long-day conditions compared to mock-treated plants. Soil water consumption was measured by gravimetric analysis, and we found that iSB09 treatment enhanced water banking (Fig. 7B). For example, 10 days after stopping irrigation, circa 70% water had been lost in pots of mock-treated plants, whereas only 40% water consumption was recorded in iSB09-treated plants (Fig. 7B). In agreement with these data, a high percentage of plants survived after iSB09 treatment (Fig. 7B). Similarly, under short-day conditions, enhanced resistance to drought was observed in iSB09-treated plants compared to mock-treated plants, as well as marked enhancement of plant survival and reduced soil water consumption (Fig. 7, C and D).

**Fig. 8.**
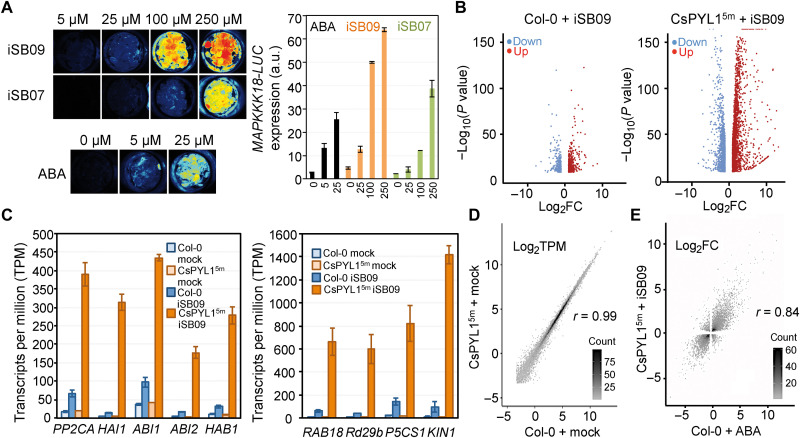
The iSB09-CsPYL1^5m^ combination strongly induces an ABA-like transcriptional response in transgenic plants. (**A**) iSB compounds induce the *pMAPKKK18-LUC* reporter line. Seedlings of the reporter line were treated with the indicated concentrations of iSB07, iSB09, or ABA in 24-well plates and imaged with a charge-coupled device camera to detect luminescence 6 hours later (left). Luciferase luminescence was quantified as indicated in Materials and Methods. (**B**) Volcano plots of RNA-seq data obtained in WT or CsPYL1^5m^ transgenic plants that were 5 μM iSB09 or mock-treated for 3 hours. Genes up-regulated (log_2_FC > 1) or down-regulated (log_2_FC < −1) with *P*_adj_ < 0.05 were plotted against the negative log_10_ *P* value. Higher values of the *y* axis indicate the stronger effect of the chemical treatment. (**C**) Induction of ABA-responsive genes by iSB09 treatment is markedly higher in CsPYL1^5m^ plants than in WT. Transcripts per million (TPMs) of the RNA-seq data were plotted for some selected ABA-responsive markers. As mock treatment, we used 0.1% DMSO in both WT and CsPYL1^5m^ plants, which was compared to 5 μM iSB09 treatment. (**D**) Scatter plots represent the genome-wide gene expression (Log_2_TPM) of mock-treated WT versus CsPYL1^5m^ plants. (**E**) Scatter plot of differentially expressed genes with *P*_adj_ < 0.05 of CsPYL1^5m^ treated with iSB09 versus control compared to WT Col-0 plants treated with ABA versus control from an independent experiment (*54*). The correlation coefficient (*r*) is indicated.

The effect of iSB09 was markedly enhanced when combined with lines expressing CsPYL1^5m^; however, the compound was also effective in WT Col-0 plants when higher dosage was applied. To examine iSB09’s effect on ABA-induced transcriptional response, we used arabidopsis lines where the ABA-responsive *MAPKKK18* promoter was fused to the LUC reporter (*45*, *46*, *62*). At 100 μM iSB9, induction of LUC was approximately twofold higher than that achieved with 25 μM ABA, which suggests that iSB09 also promotes ABA transcriptional response through activation of arabidopsis ABA receptors (Fig. 8A). iSB07’s effect at 100 μM was approximately fivefold lower than iSB09 (Fig. 8A). We also compared ABA and iSB09 up-regulation of *RAB18/RD29B* gene expression in WT Col-0 and plants that express CsPYL1^5m^ (fig. S7A). Similar expression of *RAB18/RD29B* was achieved in WT plants treated with 5 μM ABA compared to CsPYL1^5m^ plants treated with 5 μM ABA (fig. S7A). The effect of iSB09 on *RAB18/RD29B* up-regulation in CsPYL1^5m^ plants was markedly higher than in WT plants, which indicates that the iSB09-CsPYL1^5m^ combination efficiently enhances ABA-like transcriptional response (fig. S7A). To obtain a global perspective on the combined iSB09-CsPYL1^5m^ transcriptional effect, we performed transcriptome RNA sequencing (RNA-seq) by using the DNB-Seq technology. We compared the iSB09 effect (versus mock treatment) in WT and CsPYL1^5m^ transgenic lines. Appreciable up-regulation and down-regulation of ABA-responsive markers by iSB09 in WT was observed; however, a marked increase (8- to 10-fold higher) was achieved when the iSB09 ligand was applied to CsPYL1^5m^ transgenic background (Fig. 8, B and C). Data analyses indicated that iSB09–up-regulated/iSB09–down-regulated genes overlap with ABA-responsive genes (Fig. 8E), whereas the transcriptome of CsPYL1^5m^ plants was similar to WT in the absence of ligand treatment (Fig. 8D).

Last, to identify the arabidopsis receptors that mediate iSB09’s effect in WT seeds/seedlings, we analyzed the agonist-induced inhibition of seedling establishment in WT or mutant strains lacking certain arabidopsis receptors (fig. S7B) (*36*). As a result, we found that arabidopsis mutants lacking functional PYR1 and PYL1 were insensitive to 10 μM iSB09, which was corroborated in the *pyr1 pyl1 pyl2* triple and *112458* sextuple mutants (fig. S7B). Moreover, at 25 μM iSB09, the *pyr1 pyl1 pyl2* mutant showed enhanced insensitivity compared to the *pyr1 pyl1* mutant, indicating that PYL2 also recognizes iSB09 in vivo. On the other hand, *pyl4 pyl5* and *pyl8 pyl9* double mutants were sensitive to iSB09 (fig. S6B). These results suggest that iSB09’s effect in seeds is mediated mostly by dimeric receptors (subfamily III). Given that iSB09 is also perceived by PYL4 and PYL5 (Fig. 3B and fig. S6C), a diminished effect of iSB09 might be expected in *pyl4 pyl5* double mutant; however, other agonists that also target PYL5, such as QB and AMF4, lack effect in *pyr1 pyl1* double mutant (*36*). This suggests that PYL4/PYL5 effect in seed is likely overtaken by dimeric receptors.

## DISCUSSION

Chemical manipulation of ABA signaling is important for abiotic and biotic stress management in agriculture and, together with genetic approaches, can provide a powerful tool to dynamically activate the pathway (*63*, *64*). The wide information available concerning ABA biosynthesis, transport, perception, and catabolism provides clear targets for chemical-dependent control of ABA response to increase plant resistance to stress, particularly drought (*64*). Important contributions for chemical manipulation of the pathway include ABA biosynthesis inhibitors, ABA receptor agonists, and antagonists. Chemical biology approaches have enabled the discovery of an important number of ABA receptor agonists, e.g., pyrabactin, QB, AMF4, cyanabactin, opabactin, and tetralone ABA analogs, able to activate the pathway (*37*, *43*, *64*). ABA receptor antagonists have also been reported, such as AS6 and PANme (*49*, *50*), or recently antabactin (*51*). Last, repurposing of the already-in-use agrochemical MD, used originally to control oomycete pathogens, has provided a second use as ABA receptor agonist. To this end, extensive mutagenesis studies of PYR1 followed by functional analysis were required to generate the hextuple PYR1 mutant, named PYR1^MANDI^, which has nanomolar sensitivity for MD (*54*).

In this work, we took advantage of structural knowledge on crop ABA receptors, docking studies, and structure-guided ligand optimization to generate iSB09. This synthetic compound displays a U-shaped conformation in the CsPYL1 binding pocket, which is shared with all sulfonamide-based agonist molecules (*45*). The structure of these compounds consists of two facing aromatic rings that are linked by a sulfonamide bridge formed by three atoms. Despite these common structural features, the variable chemical nature of aromatic rings and the linker lead to differences in their biochemical properties. Regarding the linker, swapping the positions of the SO_2_ and CH_2_ moieties in the SB linker to produce iSB07 and iSB09 (fig. S5A) renders a marked increase in the inhibitory activity of these molecules (Figs. 2C and 3A). The structural work provided the basis of such behavior, i.e., iSB07 and iSB09 sulfonamide linkers form additional H-bonds with respect to those observed in the SB complex. In particular, that formed between the oxygen of the SO_2_ moiety and the NH^+^ of Lys^88 ^side-chain. The ligand interaction with this highly conserved Lys residue is crucial for both ABA binding and development of high-affinity agonist molecules (*36*). This conserved Lys residue forms a salt bridge with ABA’s carboxylate in WT receptors, and for example, the mutation Lys^59^Arg in PYR1 abolishes ABA binding (*54*). Comparison of iSB09 and QB sulfonamide linkers in fig. S5A shows that QB displays the sulfo moiety centered in the linker and this prevents the formation of the central H-bond to Lys^88^. This, together with metabolic studies, might provide the basis for their different biological activities and lack of QB bioactivity in tomato (fig. S6A) (*36*). Together, our structural data revealed that differences in the chemical nature of the linkers led to subtle differences in their conformations that led to major differences in the biological activity of the compounds (fig. S6A).

iSB09 activates ABA signaling in *A. thaliana* WT Col-0 plants mostly through interaction with the dimeric PYR1, PYL1, and PYL2 and monomeric PYL4 and PYL5 receptors. iSB09 was also effective to reduce transpiration in *N. benthamiana* and *S. lycopersicum* leaves, which suggests that functionally analogous receptors occur in these plant species that can be activated by iSB09. Using the available structural information, we have designed the synthetic CsPYL1^5m^ receptor to enhance the effect of iSB09, thus obtaining an optimized receptor-ligand pair, in other words, a receptor ad hoc to bind iSB09. As a result, the combined chemical-genetic approach, iSB09 + CsPYL1^5m^, was effective to enhance drought resistance in arabidopsis and we demonstrated that crop ABA receptors can be tailored to enhance the binding of agrochemicals.

The combined use of the CsPYL1^5m^ receptor–iSB09 chemical module presents a number of advantages. First, we selected a dimeric receptor and designed a series of mutations that increased its affinity for the synthetic ligand while reducing the affinity for ABA. Therefore, we did not observe constitutive activation of ABA signaling in vivo, which otherwise could be detrimental in the absence of stress (*40*, *41*). Even we could measure in vitro a reduction in ABA sensitivity of CsPYL1^5m^ compared to WT (fig. S3). Second, iSB09 showed a powerful antitranspirant effect in drought experiments, either under short-day growth chamber conditions or under long-day greenhouse conditions. Third, it is a dynamic approach, which enables flexibility in the timing and intensity of the application. Fourth, persistence of the iSB09 molecule can be expected because it is not an endogenous molecule that might follow pre-established catabolic pathways as ABA. Application of a relatively low dosage of iSB09 (5 μM) lasted at least for 48 hours to induce reduction of stomatal conductance in CsPYL1^5m^ plants (Fig. 6). Thus, in transgenic plants, a lower dosage of the chemical was required compared to nontransgenic plants. Therefore, the CsPYL1^5m^/iSB09 module would be safer from an ecological perspective focused on the limited use of agrochemicals. Last, the chemical synthesis of iSB09 is very simple, cheap, and easily scalable.

Given that ABA receptors can be functionally exchanged between different plant species (*40*, *54*), we suggest that the use of the orthogonal module formed by CsPYL1^5m^ and the iSB09 molecule could be effective to enhance drought resistance in crops. Translation of this approach to important crops is underway, and studies will be required to fully evaluate its capacity to enhance drought resistance in field conditions.

## MATERIALS AND METHODS

### Virtual screening

A subset of 140,000 molecules of the ZINC database (https://zinc.docking.org/) was used for virtual screen against ABA receptor structure (PDB code: 5MN0) using GOLD 5.4.1 (Genetic Optimisation for Ligand Docking) software (*57*). The criteria used for ZINC molecules selection included the following: being commercially available, having a molecular weight ranging from 200 to 350 Da, and containing a maximum of two hydrogen bond donor atoms and the number of hydrogen bond acceptors in the range of one to five. An initial docking was performed using the fast genetic algorithm search option; the protein was treated as rigid, and full flexibility was allowed for the ligands; the binding site was defined as the residues with at least one heavy atom within 6 Å from the ABA reference ligand placement; early termination was not allowed; and four water molecules in the active site were introduced at toggle state (the rutting chose to switch them on or off), with free rotation and displacement up to 0.5 A from their starting positions. GOLD software (using the default CHEMPLP scoring function) automatically scores the resulting ligand solutions and selects those complexes that represent potentially meaningful interactions. Besides this GOLD score, the total obtained docking poses were filtered on the basis of the results of four sphere volume descriptors, defined and evaluated using Goldmine software (*57*). Two sphere descriptors for hydrogen bond acceptors were defined as follows: one at the keto oxygen of ABA ligand in the CsPYL1-ABA-AtHAB1ΔN complex (5MN0) and the other at the carboxylate positions of that ligand with a sphere radius of 1.5 and 2.8 Å, respectively. The other two were hydrophobic sphere descriptors with a radius of 1.4 and 3.5 Å; the former was centered at the methyl group in ABA and the latter on the centroid between residues F90 and I139. When you apply/calculate these descriptors to all the ligand solutions, the volume percentage of sphere (for every defined descriptor) occupied by atoms with the required properties (in our case hydrogen bond acceptors or hydrophobic atoms) is assigned to each ligand pose. Therefore, the highest scores represent the ligand solutions that fit better our requirements. A combination of these results with the CHEMPLP ranking was the criterion that we used to select the best 93 ligands.

These 93 selected molecules were reevaluated in a more accurate genetic algorithm (GA) using 100,000 operations and 30 docking outputs, early termination was not allowed, the four water molecules were kept with toggle state as before, and 10 protein side-chain residues (K88, F90, V110, V112, L116, I139, H144, Y149, F188, and W385 from the HAB1 phosphatase) were treated as flexible with free torsional rotation. A visual inspection of the results allowed us to select the five best ligands that corroborated the important contacts and charge distribution in our experience.

Last, we purchased from MolPort Inc. the following five compounds (fig. S1): 6-methyl-7-[2-(4-methylphenyl)-2-oxoethoxy]-1*H*,2*H*,3*H*-cyclopenta[c]chromen-4-one; ({6-methyl-4-oxo-1*H*,2*H*,3*H*-cyclopenta[c]chromen-7-yl}oxy)(phenyl)acetic acid; butyl 2-({4-methyl-6-oxo-7*H*,8*H*,9*H*,10*H*-cyclohexa[c]chromen-3-yl}oxy)acetate; 5-methoxy-1-(4-methoxyphenyl)-2-methylindole-3-carboxylic acid; and *N*-benzyl-1,4-dimethyl-2-oxoquinoline-6-sulfonamide. Only the last one was able to activate the CsPYL1 receptor to inhibit HAB1 phosphatase activity.

### Protein expression and purification

Expression and purification of the sweet orange receptor CsPYL1 (amino acids 1 to 235), the quintuple mutant CsPYL1^5m^, and the phosphatase AtHAB1ΔN (amino acids 179 to 511) were done as described previously (*7*).

### Chemical synthesis

A full description of the synthesis of compounds iSB07, iSB09, and SB-01 to SB-06, as well as their analytical data, is provided in the Supplementary Materials.

### Crystallization, diffraction data collection, and structure solution

SB, iSB07, iSB09, and QB were dissolved in 40% DMSO, 30 mM tris-HCl (pH 8.5), and 6% polyethylene glycol (PEG) 400. ABA was dissolved in 2% DMSO, 50 mM tris-HCl (pH 8.5), and 10% PEG 400. Equal volumes of the solution containing ligand and a solution containing AtHAB1ΔN (5 mg/ml) and CsPYL1 or CsPYL1^5M^ (3 mg/ml) were mixed to obtain a 1:1.4:10 ratio of CsPYL1:AtHAB1ΔN:ABA or a 1:1.4:10 ratio of CsPYL1:AtHAB1ΔN:ligand. These mixtures were incubated during 1 hour at 4°C before the crystallization experiments. Crystallization of the complexes was carried out using microbath under paraffin oil technique at 18°C on a 60-well Terasaki plate (Jena Bioscience).

The best x-ray data collected for each complex correspond to crystal growth after 4 to 8 days at the following conditions: CsPYL1-SB-AtHAB1ΔN (30% PEG 3350, pH 7.5, ratio 1:2), CsPYL1^5m^-SB-AtHAB1ΔN (30% PEG 3350, pH 6.5, ratio 1:1), CsPYL1-QB-AtHAB1ΔN (25% PEG 3350, pH 6.5, ratio 1:1), CsPYL1^5m^-ABA-AtHAB1ΔN (30% PEG 3350, pH 6.0, ratio 1:1), CsPYL1-iSB07-AtHAB1ΔN (30% PEG 3350, pH 6.5, ratio 1:1), CsPYL1^5m^-iSB07-AtHAB1ΔN (30% PEG 3350, pH 6.0, ratio 1:1), CsPYL1-iSB09-AtHAB1ΔN (35% PEG 3350, pH 6.5, ratio 1:1), and CsPYL1^5m^-iSB09-AtHAB1ΔN (35% PEG 3350, pH 6.5, ratio 1:2). All crystals were cryo-protected in their corresponding crystallization solution, which also contained 30% glycerol, and flash-frozen in liquid nitrogen.

Diffraction data were collected at 100 K at the ALBA synchrotron radiation source (BL13 beamline) and processed with XDS (*65*).

The structures of the complexes were solved by molecular replacement with Phaser as a part of the Phenix suite of programs (*66*) and using as a search model the ligand-free protein structure (PDB ID: 5mn0). Refinement was performed by running several cycles of automated refinement with Phenix (*66*) followed by manual model building with Coot (*67*). The ABA and QB dictionary with geometrical restraints was first generated with the eLBOW program from the Phenix package (*66*), and later on, the dictionary was improved using the information included in the CSD (*60*). The iSB07 and iSB09 dictionaries were generated using the Grade Web Server (http://grade.globalphasing.org) run on mol2 files from the x-ray structures of the protein-free small molecules. Details on data processing and refinement are shown in table S1. SB, iSB07, and iSB09 crystal structures were solved by direct methods using SIR2011 software (*68*), and refinement was performed with SHELXL (*69*). Analysis of the structures was done with CCP4 (*70*) programs and the CSD (*60*). Images were drawn with PyMOL (*71*) and Mercury programs (*60*).

### Generation of *A. thaliana* transgenic plants

*A. thaliana* plants were grown and transformed as described (*39*). The *pAlligator2-35S:HA-CsPYL1* and *pAlligator2-35S:HA-CsPYL1^5m^* constructs were transferred to *Agrobacterium tumefaciens* C58C1 (pGV2260) by electroporation and used to transform WT Col-0 plants by the floral dip method (*39*). T1 transgenic seeds were selected on the basis of green fluorescent protein (pAlligator2) and sowed in soil to obtain the T2 generation. Homozygous T3 progeny was used for further studies, and expression of hemagglutinin (HA)–tagged protein was verified by immunoblot analysis using anti-HA–horseradish peroxidase (HRP) antibodies.

### Whole-plant gas exchange experiments

*A. thaliana* seeds were planted in specific gas exchange pots, where above- and below-ground parts can be separated by glass into 2:1 (v:v) peat:vermiculite mixture. The plants were grown in growth chambers (Snijders Scientific, Drogenbos, Belgium) at 12/12 photoperiod, 23/18°C temperature, 160 μmol m^−2^ s^−1^ light, and 70% relative humidity and were 23 to 28 days old during gas exchange experiments. Whole-rosette stomatal conductances were recorded with an eight-chamber custom-built temperature-controlled gas exchange device as described before (*72*). Plants were inserted into the measurement cuvettes and allowed to stabilize at standard conditions: ambient CO_2_, ~420 ppm; air temperature, 24° ± 0.5°C; light, 160 μmol m^−2^ s^−1^; relative air humidity, 66 ± 3%. For sprayings, plants were removed from gas exchange cuvettes, sprayed with respective solutions, and put back into the cuvettes for stomatal conductance recordings for 56 min. To check the long-term effect of compounds on plants, stomatal conductance measurements were performed about 24 and 48 hours after sprayings. In the meantime, plants were kept in growth chambers as described above. Photographs of plants were taken after the experiment, and leaf rosette area was calculated using ImageJ 1.37v [National Institutes of Health (NIH), USA]. Stomatal conductance for water vapor was calculated with a custom-written program as described (*72*).

### PP2C inhibition assays

His-tagged receptors and phosphatases were purified using nickel–nitrilotriacetic acid (Ni-NTA) affinity chromatography. Protein integrity was analyzed by SDS–polyacrylamide gel electrophoresis, followed by InstantBlue staining. Phosphatase activity was measured using p-Nitrophenyl Phosphate (pNPP) (25 mM) as substrate, 10 mM MnCl_2_,1 μM PP2C, and 2 μM of the indicated receptors. Proteins were incubated with the ligands for 10 min at room temperature. pNPP was then added to start the reactions, and the absorbance at 405 nM was monitored for 20 min in a ViktorX5 plate reader. Assays were repeated twice. For dose-response experiments, IC_50_ values were obtained with GraphPad Prism 9 using a nonlinear regression curve fit. Each ligand concentration was tested in triplicate. Phosphatase activity of ABI1 was measured using RRA(phosphoT)VA peptide as a substrate, as described (*27*).

### IR thermography

*A. thaliana* plants were grown in a controlled environment growth chamber at 21°/19°C under 8-hour light/16-hour dark photoperiod at 100 μE m^−2^ s^−1^. IR thermography images of rosette leaves were acquired with a thermal camera FLIR E95 equipped with a 42° lens, 24 hours after spraying 6-week-old plants with 10 mM MES (pH 5.7) + 0.02% Silwet L-77 plus 50 μM ligand or 0.1% DMSO as a control. Images were processed and quantified with the FLIR tools software. The average temperature of 15 different sections taken from four to six leaves per plant was used for quantification. At least six plants per genotype were analyzed, and the experiments were repeated twice. Statistical analysis was performed using GraphPad Prism 9. *T* test or ANOVA analysis was carried out to identify statistical significance among datasets.

For experiments using *N. benthamiana* or *S. lycopersium*, 1-month-old plants grown under long-day conditions (16-hour light/8-hour dark) were sprayed with 10 mM MES (pH 5.7) + 0.02% Silwet L77 supplemented with either 50 μM ABA, or the indicated concentration of iSB07/iSB09 or 0.1% DMSO as a mock-treated control. IR images were taken using a FLIR E95 thermocamera 1 day after the treatment (for *N. benthamiana*) or 2 and 5 days (for *S. lycopersicum*). Quantification was performed using the FLIR tools software on fully expanded leaves by analyzing 10 sections per leaf. At least six plants per genotype and treatment were analyzed in each experiment. Experiments were repeated twice. The average plant temperature ± SD of all the plants for each treatment and genotype was calculated and used to report the increase in temperature produced by each treatment. Statistical comparisons among genotypes were performed by either pairwise *t* tests or one-way ANOVA.

### Root growth assays

Seedlings were grown on vertically oriented Murashige and Skoog (MS) plates for 4 to 5 days. Afterward, seedlings were transferred to MS supplemented with the indicated concentrations of the different ligands and kept on vertical orientation. Ten days later, the plates were scanned on a flatbed scanner to produce image files suitable for quantitative analysis of root growth using the NIH Image software ImageJ. The experiment was performed in triplicate (*n* = 13 to 16). Statistical comparisons were performed by either pairwise *t* tests or one-way ANOVA using GraphPad Prism 9.

### Drought resistance experiments

For drought assays under long-day conditions, seeds from WT Col-0 and *pAlligator2-35S:CsPYL1^5m^* plants were grown in MS medium for 7 days. Then, seedlings were transferred to individual 0.18-liter pots containing equal amount of moisted soil composed of peat, vermiculite, and perlite at 1:0.5:0.5 ratio (v/v). Plants were grown under long-day conditions in a controlled environment growth chamber at 22°C under 16-hour light/8-hour dark photoperiod under well-watered conditions for 3 weeks. Then, watering was withheld for 14 days. Plants were treated by spraying with a solution containing 10 mM MES (pH 5.7), 0.02% Silwet L77, and either 0.1% DMSO (control), 50 μM ABA, or iSB09 twice. The first application was performed the same day the watering was withheld, and the second one was performed 7 days later. Watering was restored and survival rate was measured 6 days after rewatering. Gravimetric analysis of water loss in pots was performed along the experiment.

For drought assays under short-day conditions (8-hour light/16-hour dark photoperiod), 4-week-old plants were deprived of water and treated with 10 mM MES (pH 5.7) + 0.02% Silwet L77 and either 0.1% DMSO (control), 50 μM ABA, or iSB09 every 7 days. Pots were weighted at 7-day intervals. Pictures of the plants were taken at the beginning of the experiment, 26 days after water deprivation and 12 days after rewatering. Survival rate and foliar leaf area were measured 12 days after rewatering.

For drought experiments, 6 to 10 plants per genotype and treatment were analyzed in each experiment. The experiment was performed twice.

### Seed germination and seedling establishment assays

For seed germination assays, seeds were stratified in the dark at 4°C for 3 days. Approximately 100 seeds of each genotype were sown on MS plates supplemented with six different ligand concentrations. The experiments were repeated three times. To score seed germination, radical emergence was analyzed at 24 hours after sowing.

Seedling establishment was scored as the percentage of seeds that developed green expanded cotyledons and the first pair of true leaves at 5 or 7 days. Seedling establishment assays in the presence of ABA receptor agonists were performed in 24-well plates, where approximately 25 to 35 seeds per well were sown. Each well contained a different treatment. The experiment was repeated at least twice.

### Luciferase experiments

*pMAPKKK18-LUC* seedlings were grown in 24-well plates (25 to 35 seeds per well) filled with 1 ml of liquid MS medium for 7 days. MS medium was replaced with fresh medium supplemented with 100 μM d-luciferin, potassium salt (GoldBio), and the indicated treatments with iSB07, iSB09, or ABA. Seedlings were incubated for 6 hours, and luminescence was recorded with a LAS-3000 imager (Fujifilm) equipped with a charge-coupled device camera using 2-min exposures. Eight-bit images were transformed in rainbow false color and quantified using Fiji. The experiment was repeated at least twice.

### Reverse transcription quantitative polymerase chain reaction

RNA was extracted from seedlings using NucleoSpin RNA Plant kit from Machery-Nagel, following the manufacturer’s instructions. Complementary DNA (cDNA) was synthesized from 1 μg of total purified RNA using 30 U of RevertAid Reverse Transcriptase (Thermo Fisher Scientific). Reverse transcription quantitative polymerase chain reaction (RT-qPCR) was performed using PyroTaq EvaGreen qPCR Master Mix 5X from Cultek, which includes EvaGreen Dye and carboxy-X-rhodamine (ROX) as a passive reference dye. The primer pairs used for this analysis were described previously (*42*). qPCR was performed in a QuantStudio 3 (Applied Biosystems). Relative quantification of gene expression data was carried out using the 2^−ΔΔCt^ or comparative Ct method. Expression levels were normalized using the Ct values obtained for the actin-8 gene. The presence of a single PCR product was further verified by dissociation analysis in all amplifications. The mean and SD of three independent experiments are shown.

### RNA sequencing

Ten-day-old WT or CsPYL1^5m^ seedlings were mock- or iSB09 (5 μM)–treated, and samples were collected after 3 hours (three independent experiments). Total RNA was extracted as indicated above. The quality of the samples was verified using Agilent 2100 Bioanalyzer. First-strand cDNA was prepared by an EasyScript One-Step gDNA Removal and cDNA Synthesis SuperMix (TransGen Biotech) with random hexamers. Sequencing of cDNA libraries was performed using the DNB-Seq technology at the Beijing Genomics Institute, producing at least 20 million clean reads per sample of paired-end 100–base pair read lengths. RNA-seq data analysis was carried out by the Bioinformatics core facility at Instituto de Biología Molecular y Celular de Plantas. After quality analysis of raw reads with FastQC (www.bioinformatics.babraham.ac.uk/projects/fastqc/), and quality trimming and adapter removal of raw reads with cutadapt (http://journal.embnet.org/index.php/embnetjournal/article/view/200), clean read pairs longer than 20 nucleotides were mapped to TAIR10 arabidopsis Col-0 reference genome using HISAT2 with default parameters, and nonuniquely mapped pairs were discarded with SAMtools.

The number of read counts that uniquely mapped to each arabidopsis gene (Araport11 annotation) was obtained with HTSeq-count. Genes lacking at least 1 TPM (transcripts per kilobase of exon model per million mapped reads) in the three replicates of at least one of the two conditions were filtered out, and differential expression analysis of the remaining genes was done with DESeq2. Genes with an absolute value of log_2_ fold change (log_2_FC) > 1 and *P*-adjusted value (*P*_adj_) < 0.05 were considered differentially expressed genes. Raw reads and differential expression gene tables used here have been deposited in the Gene Expression Omnibus under accession no. GSE193570. Volcano plots were constructed using the EnhancedVolcano R package. The RNA-seq datasets from ABA-treated seedlings (*54*) were filtered by absolute log_2_FC > 1 and *P*_adj_ < 0.05 and used to compare with our RNA-seq data.

### Isothermal titration calorimetry

ITC measurements were performed at 35°C with an Auto-iTC200 isothermal titration calorimeter (Micro-Cal-Malvern) in a buffer consisting of 50 mM Hepes (pH 7.5), 200 mM NaCl, 5 mM MgCl_2_, 10% glycerol, 1 mM β-mercaptoethanol, and 0.2% DMSO. For binding measurements, a 1:1 mixture of receptor:ΔNHAB1 (20 μM) was titrated with a solution of 200 μM ABA or iSB09 as corresponds. To obtain the *K*_d_ values, the data were analyzed using nonlinear least-squares regression using a model considering a single set of binding sites implemented in Origin 7.0 (OriginLab).

### Native red electrophoresis

A 1:1 mixture of receptor:ΔNHAB1 (18 μM) was mixed with increasing concentrations of ABA or iSB09, as indicated in Fig. 3 (E and F), in a buffer consisting of 50 mM tris-HCl (pH 7.5), 100 mM NaCl, 5 mM MgCl_2_, 5 mM dithiothreitol (DTT), 10% glycerol, and 0.02% Ponceau Red S. Samples were incubated during 30 min at room temperature and were loaded onto a 13.5% polyacrylamide gel prepared in 375 mM tris-HCl (pH 8.8), 10% glycerol, and 0.012% of Ponceau Red S. A solution of tris-glycine (pH 8.8) with or without 3 mM MgCl_2_ and 0.012% Ponceau Red S was used as the cathode and anode buffer, respectively. Electrophoresis was performed at a constant current of 25 mA for 150 min at 4°C. Proteins were detected in gel by InstantBlue staining, and band quantification was performed using the ImageJ software. To obtain apparent *K*_d_ values, the proportion of ternary complex formed (fraction bound) and the ABA or iSB09 free concentration in each lane were calculated and plotted as represented in Fig. 3 (E and F). Then, the dose-response curves were fitted to the classical Hill equation using the GraphPad Prism software.

## Notes

View/request a protocol for this paper from *Bio-protocol*.
