# Supplementary material for: Structure-guided engineering of a receptor-agonist pair for inducible activation of the ABA adaptive response to drought
Source: Sci Adv. 2023 Mar 10;9(10):eade9948. doi: 10.1126/sciadv.ade9948 (PMC10005185; doi:10.1126/sciadv.ade9948)
Supplement: Supplementary file 1 — Supplementary Text Figs. S1 to S7 Table S1 [file sciadv.ade9948_sm.pdf]

Science Advances
AAAS

Supplementary Materials for

Structure-guided engineering of a receptor-agonist pair for inducible activation of the ABA adaptive response to drought

Jorge Lozano-Juste *et al.*

Corresponding author: Pedro L. Rodriguez, prodriguez@ibmcp.upv.es; Armando Albert, xalbert@iqfr.csic.es

Sci. Adv. **9**, eade9948 (2023)
DOI: 10.1126/sciadv.ade9948

This PDF file includes:

Supplementary Text
Figs. S1 to S7
Table S1

Supplementary Text

Chemical synthesis

SB was purchased from UAB Crea-Chim (Lithuania). Synthesis of iSB07 and iSB09, as well as SB derivatives that maintain the same sulfonamide arrangement as SB are described in Supplemental information. All reactions were carried out under air unless stated otherwise. Reactions were monitored by thin-layer chromatography (TLC) analysis on Merck® silica gel 60 F254 TLC plates. Spots were visualized by exposure to ultraviolet (UV) light (254 nm), or by staining with a 5% solution of phosphomolybdenic acid (PMA) in ethanol or basic aqueous potassium permanganate ($KMnO_4$) and then heating. Flash chromatography was carried out using Merck® silica gel 60 (230-400 mesh). All solvents were of HPLC grade quality and used as received. All reagents were purchased at the highest commercial quality and used without further purification. 1H-NMR spectra were recorded on a Varian Mercury (300 MHz) spectrometer. Chemical shifts are reported in parts per million (ppm) down field from TMS as an internal standard. Data are reported as follows: chemical shift, multiplicity (s=singlet, d=doublet, t=triplet, q=quartet, dd=doublets of doublets, m=multiplet, br=broad), coupling constant(s) and integration. HPLC analyses were carried out on a Waters system model Alliance HT (Mass detector: Micromass ZQ 2000). A: water, B: CH_3CN:CH_3OH (1:1), C: 100 mM ammonium acetate solution (approx. pH 6.8). Method A (9 min): Analysis conditions: Luna C18(2) 5 µm, 2.0 × 50 mm. Gradient: A:B:C 30 s at 85:10:5; then from 85:10:5 to 0:95:5 in 4 min, finally 4.5 min at 0:95:5. Method B (15 min): Analysis conditions: Luna C18(2) 5 µm, 2.0 × 50 mm. Gradient: A:B:C 3 min at 85:10:5; then from 85:10:5 to 0:95:5 in 6 min, finally 7 min at 0:95:5. Method C (30 min): Analysis conditions: SunFire C18 3.5 µm, 2.1 × 100 mm.Gradient: A:B:C 5 min at 85:10:5; then from 85:10:5 to 0:95:5 in 15 min, finally 10 min at 0:95:5.

Synthesis of iSB07 and iSB09:

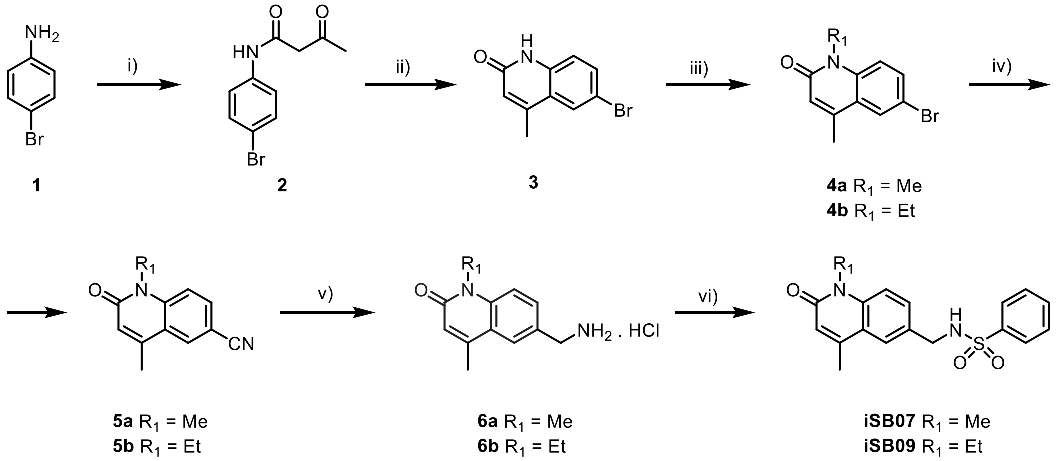

i) Ethyl acetoacetate, xylene, 135 ºC, 5 h; ii) H_2SO_4, 100 ºC, 2 h; iii) R_1I, NaH, rt, 4-6 h; iv) $Zn(CN)_2$, $Pd(PPh_3)_4$, DMF, 100 ºC, 6 h; v) H_2, Pd-C, HCl (37%), MeOH, rt, 1-2 days; vi) $PhSO_2Cl$, DIPEA, DMF, rt, 2 h.

i) *para*-Bromoaniline (**1**, 52,31 mmol) was dissolved in xylene (30 mL) and ethyl acetoacetate was added (1.2 equiv). The solution was heated at 135 °C for 24 h. After allowing the flask to naturally cool down to room temperature (rt), the reaction flask was placed in the freezer for 5 h, obtaining a white precipitate. The solid was filtered off, washed with hexane (3 x 20 mL) and dried under vacuum to afford **2** as a light brown solid (22% isolated yield). LC-MS (Method B): Purity=96.37%, M+1=254.1 (ESI-).

ii) **2** (10.5 mmol) was dissolved in H_2SO_4 (10 mL, 97%) and the solution was heated at 100 °C for 2 h. After cooling down to rt, iced water was added dropwise (total volume of 20 mL). The resulting suspension was left stirring overnight at rt. The white solid was filtered off, washed with water (3 x 3 mL), Et_2O (5 x 3 mL), and finally with hexane (3 x 3 mL), affording **3** as a grey-ish solid (92% isolated yield). LC-MS (Method A): Purity=91.0%, M+1=239.1 (ESI+).

iii) **3** (1,68 mmol) was suspended in DMF (25 mL), affording a grey-ish suspension. To this mixture was added NaH (60%, 3 equiv.) at rt, after which bubbling was observed. The reaction mixture was left at rt for 20 min. Next, the corresponding alkylation reagent (MeI or EtI) was added (1 equiv). The reaction was completed in 4-6 h, after which the solution was concentrated under vacuum, the residue was dissolved in DCM (15 mL), and water (20 mL) was added. The layers were separated, and the aqueous phase was further washed with DCM (2 x 15 mL). The combined organic layers were dried over Na_2SO4, filtered off, and the solvent removed under vacuum, affording a light brown solid in the case of **4a** (63% isolated yield; LC-MS (Method B): Purity=98.68%, M+1=252.1 (ESI+)) and a yellow-ish solid in the case of **4b** (82% isolated yield; LC-MS (Method B): Purity=96.33%, M+1=265.8 (ESI+)).

iv) **4** (1.31 mmol) was dissolved in DMF and then $Zn(CN)_2$ (2 equiv) was added. The mixture was deoxygenated for 5 min with nitrogen, before adding $Pd(PPh_3)_4$ (0.1 equiv). The reaction was heated at 100 °C until completion (2-6 h). The mixture was allowed to cool down to rt and poured over an aqueous solution of sat. NaCl. This mixture was extracted with AcOEt (3 x 10 mL) and the combined organic layers were dried over Na_2SO_4, filtered off, and the solvent removed under vacuum. The resulting residue was washed with hexane (2 x 1 mL) and Et_2O (2 x 1 mL) to afford **5a** as a white solid (64% isolated yield; LC-MS (Method B): Purity=98.87%, no ionization) or **5b** as a yellow-ish solid (91% isolated yield; LC-MS (Method B): Purity=98.94%, M+1=213.2 (ESI+)).

v) **5** (1.18 mmol) was suspended in MeOH (10 mL) and HCl (37%, 3 mL) was added. The mixture was bubbled with N_2 for 5 min to remove oxygen, after which the catalyst Pd-C (10%, 0.1% wt) was added. The reaction mixture was placed under atmospheric pressure of H_2 (rubber balloon) and left stirring at rt until completion (1-2 days). Next, the mixture was filtered through Celite® with MeOH washes (4 x 5 mL). The solvent was removed under vacuum affording **6a** as a white solid (98% isolated yield; LC-MS (Method B): Purity= 96.99%, M+1= 203.0 (ESI+) or **6b** as a yellow-ish solid (94% isolated yield; LC-MS (Method B): Purity=93.41%, M+1=218.1 (ESI+).

vi) **6** (1 mmol) was suspended in DMF (7 mL) and DIPEA (3 equiv) was added, affording a yellow solution. Benzenesulfonyl chloride (1.2 equiv) was then added, and the reaction was stirred for 2 h at rt. The solvent was then removed under vacuum and the resulting residue dissolved in DCM (10 mL). Aqueous NH_4Cl was added (20 mL) and the organic layer separated. The aqueous phase was further extracted with DCM (2 x 10 mL). The combined organic layers were dried over Na_2SO_4, filtered off, and the solvent removed under vacuum. The resulting oily solid was finally purified by silica column (MeOH 3%→5%/DCM) affording the desired final product.

iSB07: white solid, 52% isolated yield; LC-MS (Method C): Purity=99.15%, M+1=343.1 (ESI+). 1H NMR (300 MHz, DMSO-*d6*): δ 8.25 (s, 1H), 7.84 – 7.70 (m, 2H), 7.65 – 7.37 (m, 6H), 6.51 (d, J = 1.4 Hz, 1H), 4.12 (s, 2H), 3.56 (s, 3H), 2.34 (s, 3H).

iSB09: white solid, 45% isolated yield. LC-MS (Method C): Purity=99.82%, M+1=357.1 (ESI+). 1H NMR (300 MHz, DMSO-*d6*): δ 8.23 (s, 1H), 7.76 (dd, J = 8.0, 1.7 Hz, 2H), 7.62 – 7.43 (m, 6H), 6.49 (s, 1H), 4.23 (q, J = 7.1 Hz, 2H), 4.12 (s, 2H), 2.34 (s, 3H), 1.16 (t, J = 7.0 Hz, 3H).

Synthesis of SB derivatives:

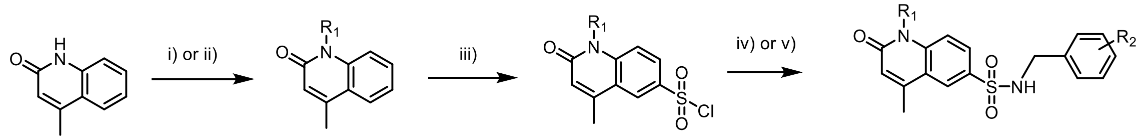

7

8a R_1 = Et
8b R_1 = cPr
8c R_1 = Me

9a R_1 = Et
9b R_1 = cPr
9c R_1 = Me

SB-01 R_1 = Et; R_2 = H
SB-02 R_1 = cPr; R_2 = H
SB-03 R_1 = Me; R_2 = *p*-Me
SB-04 R_1 = Me; R_2 = *o*-F
SB-05 R_1 = Me; R_2 = *o*-Cl
SB-06 R_1 = Me; R_2 = *o*-OH

i) R_1X, NaH, DMF, rt. 4-6 h; ii) cyclopropylboronic acid, $(AcO)_2Cu$, Pyr, NaHMDS, toluene, 100 ºC, 20 h; iii) $ClSO_3H$, 50 °C, 4-6 h; iv) $(R_2)PhCH_2NH_2$, Py, DMF, rt, 1-2 h; v) $(R_2)PhCH_2NH_2$, Et_3N, DCM, 0 ºC to rt, 4 h.

i) **7** (4.39 mmol) was suspended in DMF (10 mL) under N_2 atmosphere. To this mixture was added NaH (60%, 3 equiv.) at rt, after which bubbling was observed in the grey-ish solution. The reaction mixture was left at rt for 20 min. Next, the corresponding alkylation reagent (MeI or EtBr) was added (2.5 equiv). The reaction was completed in 4-6 h, after which it was quenched with a few drops of water. Next, NaCl (sat aq, 30 mL) was added and extracted with AcOEt (2 x 30 mL). The combined organic phases were dried over Na_2SO_4, filtered off, and the solvent removed under vacuum. The obtained crude product was finally purified by chromatography (SiO_2, 30%→60% AcOEt/Hexane).

8a: yellow oil, 70% yield. LC-MS (Method A): Purity=93.46%, M-Me=173.6 (ESI+).

8c: white solid, 89% yield. LC-MS (Method A): Purity=98.84%, M+1=199.7 (ESI+).

ii) **7** (3.76 mmol) was suspended in toluene (20 mL), after which cyclopropylboronic acid (2 equiv), $(AcO)_2Cu$ (1 equiv), and pyridine (5 equiv) were sequentially added. The mixture was deoxygenated with N_2 and NaHMDS (1 equiv) was added. The reaction mixture was stirred at 100 °C under constant air flow for 15 h. After cooling down to rt, the mixture was filtered through celite with AcOEt washes (20 mL). The organic phase was further washed with water (20 mL) and subsequently dried over Na_2SO_4, filtered off, and the solvent removed under vacuum to give the desired product **8b** as a brown oil, 89% yield. LC-MS (Method B): Purity=88.21%, M+1= 173.6 (ESI+).

iii) $ClSO_3H$ (1.5 mL) was added dropwise to **8** (1.84 mmol) at low temperature (ice bath). The mixture was then heated to 50 °C and stirred for 4-6 h. Next, the mixture was poured over crushed ice and NaCl (sat aq, 30 mL) was added. This aqueous mixture was extracted with DCM (2 x 30 mL), and the organic phase dried over Na_2SO_4, filtered off, and the solvent removed under vacuum to give the desired product.

9a: light-brown solid, 77% isolated yield after chromatography (SiO_2, 30% AcOEt/Hex). 1H NMR (300 MHz, $CDCl_3$) δ 8.34 (s, 1H), 8.16 (d, J = 9.1 Hz, 1H), 7.54 (d, J = 9.2 Hz, 1H), 6.72 (s, 1H), 4.38 (q, J = 7.2 Hz, 2H), 2.54 (s, 3H), 1.37 (t, J = 7.1 Hz, 3H).

9b: brown solid, 27% isolated yield after chromatography (SiO_2, 50% AcOEt/Hex).

9c: brown solid, 60% isolated yield after chromatography (SiO_2, 30% AcOEt/Hex). LC-MS (Method A): Purity=89.32%, M+1 (acid)=252.2 (ESI+).

iv) To a solution of **9a** or **9c** (0.73 mmol) in DMF (5 mL) was added pyridine (1.1 equiv) and the corresponding benzylamine (1.05 equiv). The resulting mixture was stirred at rt for 1-2 h. The mixture was diluted with water (15 mL) and extracted with AcOEt (20 mL). The organic phase was separated and washed successively with HCl (10%, 10 mL), $NaHCO_3$ (aq. sat, 10 ml), and NaCl (sat. aq., 10 mL), to be finally dried over Na_2SO_4, filtered off, and the solvent removed under vacuum. The residue was then washed with Et_2O or AcOEt to obtain the desired product.

SB-01: white solid, 16% isolated yield; LC-MS (Method C): Purity=99.16%, M+1=357.1 (ESI+).).1H NMR (300 MHz, $CDCl_3$) δ 8.19 (s, 1H), 8.01 (d, J = 9.0 Hz, 1H), 7.48 (d, J = 9.0 Hz, 1H), 7.27 (dd, J = 13.3, 7.6 Hz, 5H), 6.73 – 6.66 (m, 1H), 4.92 (t, J = 6.3 Hz, 1H), 4.40 (q, J = 7.1 Hz, 2H), 4.23 (d, J = 6.2 Hz, 2H), 2.50 (s, 3H), 1.45 – 1.34 (m, 3H).

SB-03 white solid, 18% isolated yield; LC-MS (Method C): Purity=96.32%, M+1=357.2 (ESI+).).^{1}H NMR (300 MHz, $CDCl_3$) δ 8.14 (s, 1H), 8.01 (dd, *J* = 8.7, 2.0 Hz, 1H), 7.46 (d, *J* = 9.0 Hz, 1H), 7.28 (s, 1H), 7.06 (s, 4H), 6.69 (s, 1H), 4.84 (s, 1H), 4.16 (d, *J* = 5.6 Hz, 2H), 3.75 (s, 3H), 2.48 (s, 3H), 2.29 (s, 3H).

SB-04: beige solid, 9% isolated yield; LC-MS (Method C): Purity=95.12%, M+1=361.0 (ESI+).). ^{1}H NMR (300 MHz, $CDCl_3$) δ 8.09 (d, *J* = 2.3 Hz, 1H), 7.94 (dd, *J* = 9.0, 2.3 Hz, 1H), 7.37 (d, *J* = 8.6 Hz, 1H), 7.30 – 7.09 (m, 2H), 7.04 – 6.93 (m, 1H), 6.86 (dd, *J* = 10.5, 8.0 Hz, 1H), 6.65 (s, 1H), 5.03 (s, 1H), 4.27 (d, *J* = 6.3 Hz, 2H), 3.70 (d, *J* = 1.3 Hz, 3H), 2.45 (q, *J* = 1.3 Hz, 2H).

SB-05: off-white solid, 33% isolated yield; LC-MS (Method C): Purity=97.28%, M+1=377.0 (ESI+).).^{1}H NMR (300 MHz, $CDCl_3$) δ 8.09 (d, *J* = 2.1 Hz, 1H), 7.95 (dd, *J* = 8.9, 2.1 Hz, 1H), 7.38 (d, *J* = 8.9 Hz, 1H), 7.31 – 7.24 (m, 2H), 7.21 (d, *J* = 4.1 Hz, 1H), 7.18 – 7.08 (m, 2H), 6.67 (s, 1H), 5.15 (t, *J* = 6.4 Hz, 1H), 4.33 (d, *J* = 6.4 Hz, 2H), 3.72 (s, 3H), 2.47 (s, 3H).

v) A solution of **9b** or **9c** (1.04 mmol) in DCM (10 mL) was cooled down in an ice bath, after which Et_3N (1.1 equiv) and the corresponding benzylamine (1.05 equiv) were added. The reaction mixture was stirred at low temperature for 1-3 h (until completion). The mixture was diluted with water (15 mL) and extracted with DCM (10 mL). The organic phase was separated and washed successively with HCl (10%, 10 mL), $NaHCO_3$ (sat. aq., 10 mL), and NaCl (sat, 10 mL), to be finally dried over Na_2SO_4, filtered off, and the solvent removed under vacuum. The obtained residue was washed several times with Et_2O and AcOEt. The crude solid was further purified by chromatography to obtain the desired product.

SB-02: white solid, 4% isolated yield after purification by reverse phase chromatography (C18, MeCN/buffer pH7 65%→85%); LC-MS (Method C): Purity=81.25% +18.55%, M+1=369.1 (ESI+). ^{1}H NMR (300 MHz, $CDCl_3$) δ 8.11 (s, 1H), 7.95 (s, 2H), 7.30 – 7.16 (m, 6H), 6.57 (s, 1H), 4.87 (s, 1H), 4.19 (d, *J* = 5.9 Hz, 2H) 2.95 (s, 1H), 2.43 (d, *J* = 3.2 Hz, 3H), 1.60 (s, 3H)1.41 (s, 2H), 0.89 (s, 2H).

SB-06: white solid, 8% isolated yield after purification by chromatography (SiO_2, 5%MeOH/DCM); LC-MS (Method C): Purity=96.51%, M+1=359.1 (ESI+). ^{1}H NMR (300 MHz, DMSO-*d6*) δ 8.10 – 8.03 (m, 1H), 8.00 – 7.90 (m, 1H), 7.65 (d, *J* = 8.9 Hz, 1H), 7.12 (d, *J* = 7.4 Hz, 1H), 6.96 (s, 1H), 6.71 – 6.60 (m, 3H), 3.91 (s, 2H), 3.61 (d, *J* = 4.1 Hz, 3H), 2.42 (s, 2H).

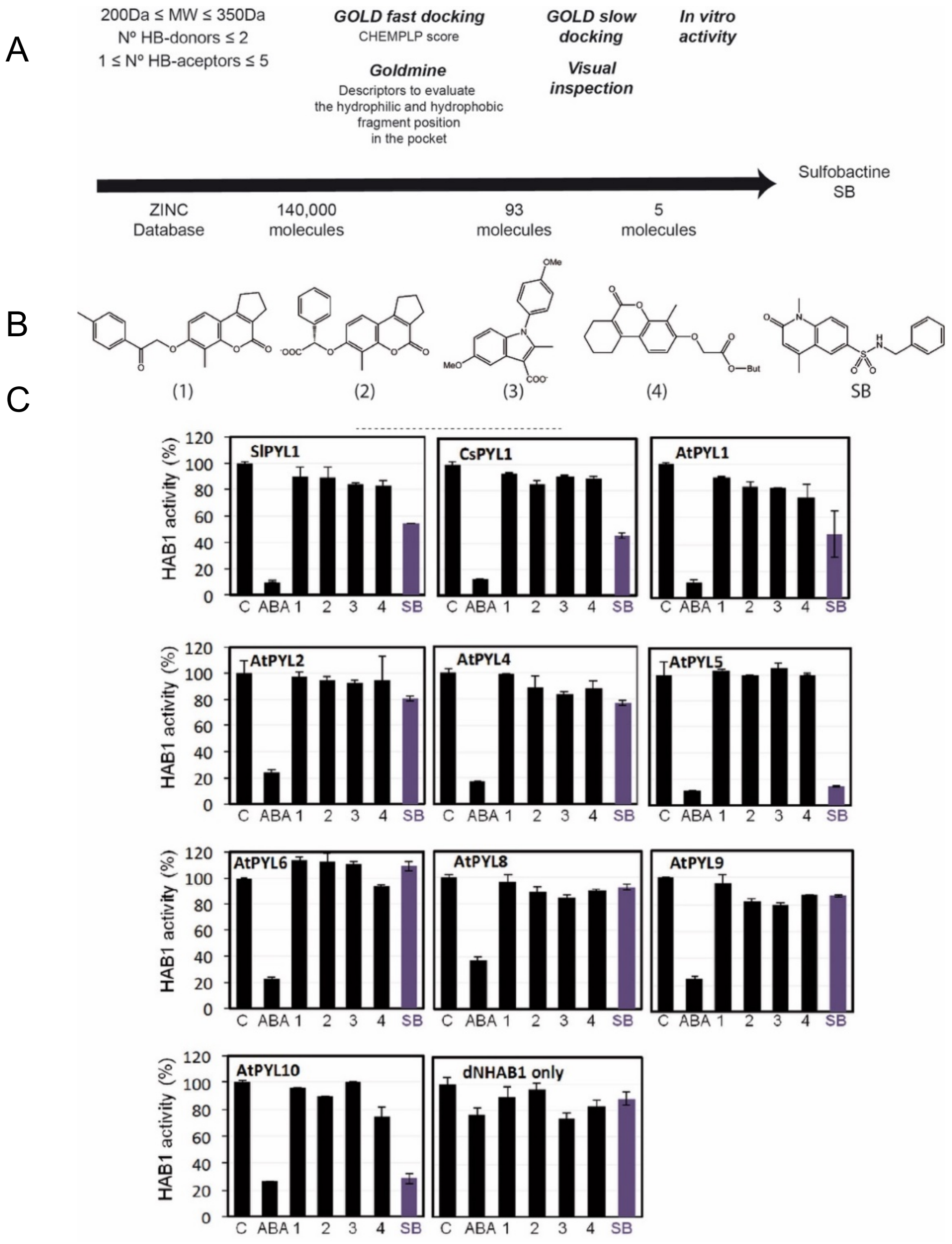

Fig. S1. **Summary of the drug-discovery approach and SB identification.**

(A) Scheme of the *in silico* and *in vitro* procedure followed for SB discovery. (B) Chemical structure of the five potential ABA agonist molecules identified using *in silico* screening: (1) (6-methyl-7-[2-(4-methylphenyl)-2-oxoethoxy]-1H,2H,3H-cyclopenta[c]chromen-4-one; (2) ({6-methyl-4-oxo-1H,2H,3H-cyclopenta[c]chromen-7-yl}oxy)(phenyl)acetic acid; (3) 5-methoxy-1-(4-methoxyphenyl)-2-methylindole-3-carboxylic acid; (4) butyl 2-({4-methyl-6-oxo-7H,8H,9H,10H-cyclohexa[c]chromen-3-yl}oxy)acetate; and (SB) N-benzyl-1,4-dimethyl-2-oxoquinoline-6-sulfonamide). (C) PP2C assays show that SB was able to inhibit HAB1 activity in the presence of AtPYL5, AtPYL10 and PYL1-like receptors, whereas the other candidate molecules were not effective

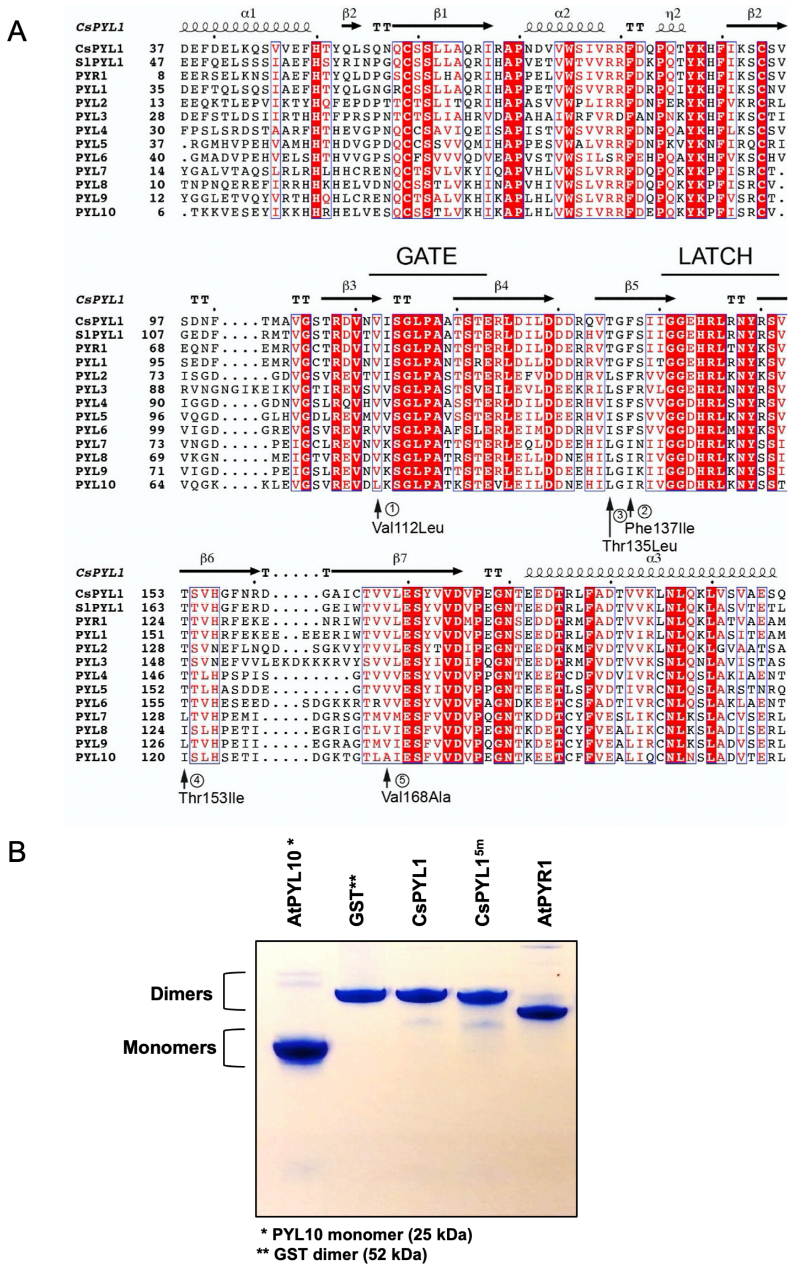

Fig. S2. The mutations engineered into CsPYL1^{5m} do not prevent dimer formation.

(A) Amino acid sequence alignment of Arabidopsis, CsPYL1 and SlPYL1 ABA receptors identifies unique changes in PYL10 that were engineered into the synthetic CsPYL1^{5m} receptor. The position of the five amino acid substitutions introduced in CsPYL1^{5m} is indicated. Alignment was generated using GeneDoc and ClustalW software. The predicted secondary structure of the receptors is indicated, taking as a model the crystallographic structure of CsPYL1 (Protein DataBank Code 5MMQ) and using the ESPRIPT program (http://espript.ibcp.fr/ESPript/ESPript) (B) Engineering of the above mutations into CsPYL1^{5m} does not affect the dimeric nature of the receptor. Native Red Elecrophoresis (NRE) analysis was performed using AtPYL10 (monomeric receptor) and dimeric GST as protein markers.

A

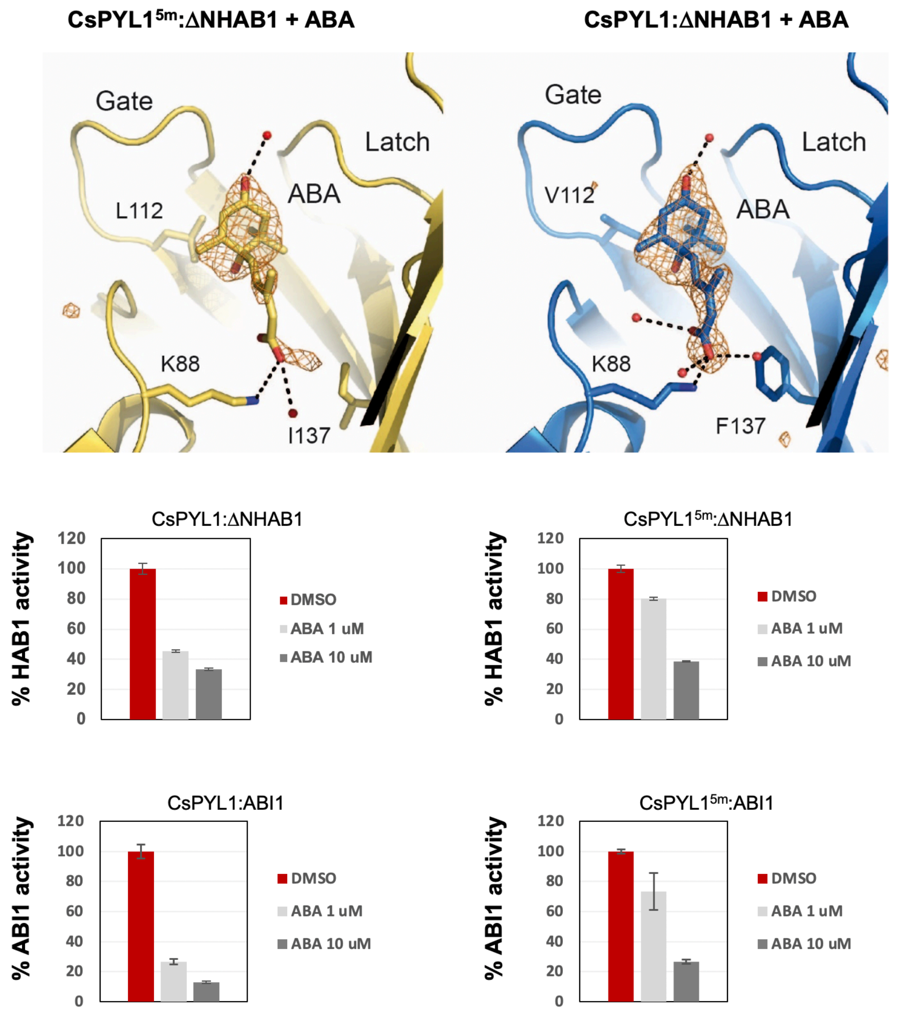

C

Protein complex	Ligand	K_D (nM)
**CsPYL1:ΔNHAB1**	ABA	500
**CsPYL1^{5m}:ΔNHAB1**	ABA	3900

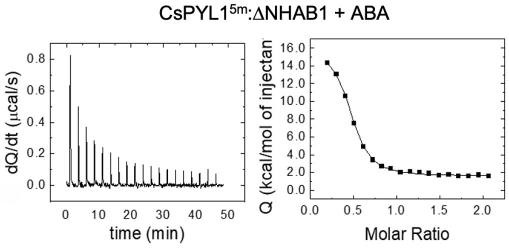

D

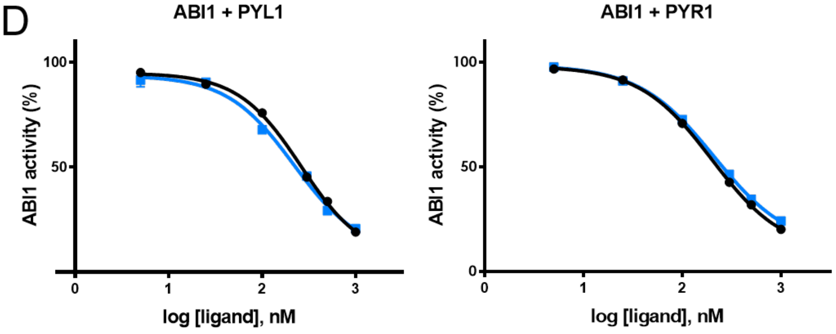

Fig. S3. CsPYL1^{5m} shows lower affinity for ABA binding than CsPYL1.

(A) A comparison of the ABA binding pocket in the CsPYL1^{5m}-ABA-AtHAB1ΔN (left) and CsPYL1-ABA-AtHAB1ΔN (right) complexes. The corresponding sections of the unbiased omit Fo-Fc maps contoured at 3s are also shown. Note a reduction of the water mediated hydrogen bonds to the receptor in the vicinity of the carboxylate group for CsPYL1^{5m} that leads to a looser binding of this moiety as shown by the weaken electron density. (B) PP2C inhibition assays (phosphopeptide as a substrate) show that ABA was less effective in CsPYL1^{5m} than CsPYL1 to inhibit HAB1 and ABI1 phosphatase activity. (C) Lower affinity of ABA for CsPYL1^{5m} than CsPYL1. ITC data were obtained by repeated injections of ABA into a 1:1 mixture of receptor: DNHAB1. (D) Dose response analysis of iSB09 or ABA-dependent inhibition of ABI1 (blue and black lines, respectively) in presence of either AtPYL1 or AtPYR1. Values (average of duplicates) show PP2C activity after incubation with the indicated concentration of ligand and receptor at 1:2 ratio (1 μM phosphatase:2 μM receptor). The IC50 of ABI1 with AtPYL1 was 274 nM and 226 nM, whereas with AtPYR1 was 200 nM and 213 nM, for ABA and iSB09, respectively. Ligands were assayed at 5, 25, 100, 300, 500 and 1000 nM.

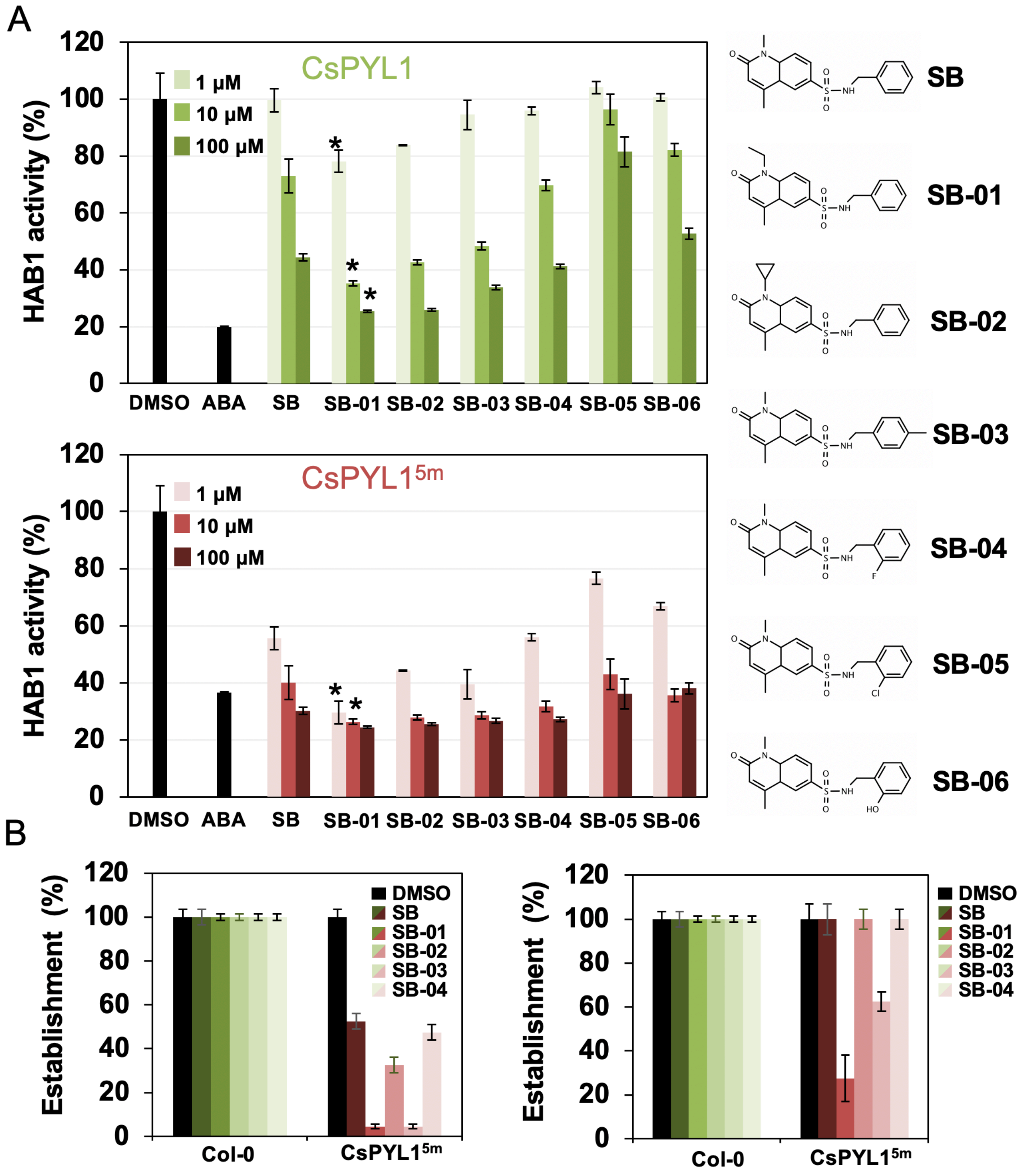

Fig. S4. In vitro and in vivo activity of SB derivatives that maintain the sulfonamide arrangement.

(A) PP2C inhibition assays show enhanced inhibition of HAB1 by SB-01 with both CsPYL1^{5m} and CsPYL1 compared to SB. * indicates $p<0.05$ (Student's t test) compared to SB at the same dosage. (B) Quantification of seedling establishment inhibition by 10 μM SB derivatives in the CsPYL1^{5m} overexpressing line compared to wildtype Col-0 at 72 h (left) or 7 d (right).

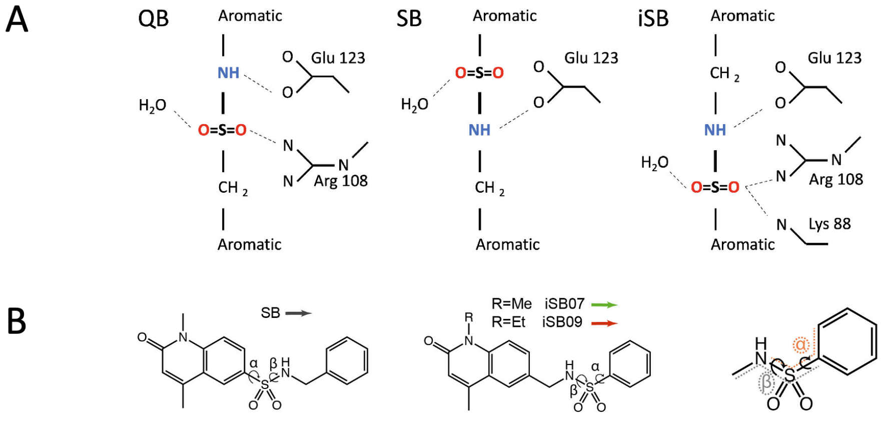

	SB_Xtal	SB_WT	SB_5M	iSB07_Xtal	iSB07_WT	iSB07_5M	iSB09_Xtal	iSB09_WT	iSB09_5M
α (°)	98.5	38	49	85.3	127	111	96.7/79.9	116	115
β (°)	-70.3	83	72	61.0	-92	-79	74.3/-66.8	-84	-83

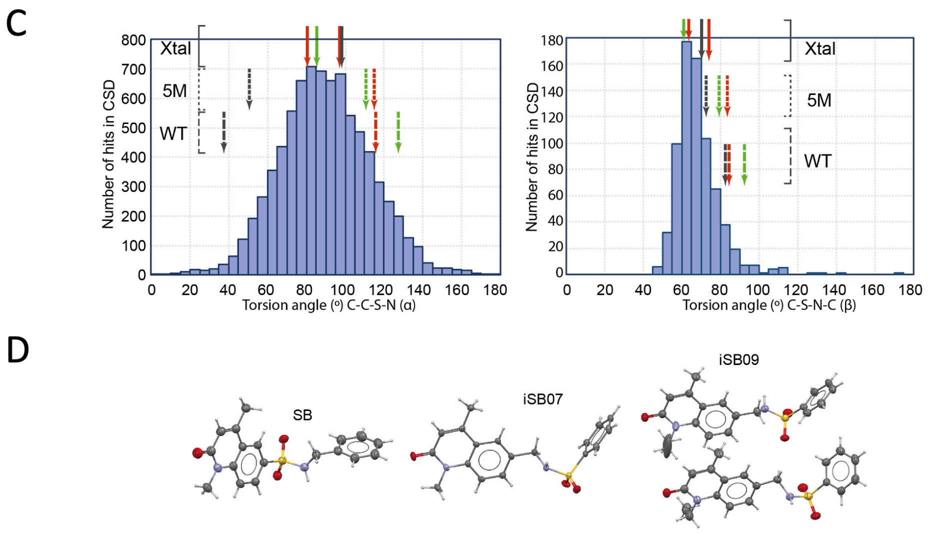

Fig. S5. H-bonds of the sulfonamide linkers and dihedral angles of different ligands.

(A) Schematic representation of the linker moieties of QB, SB and iSB09 showing their hydrogen bond interactions to CsPYL1 in the CsPYL1-ligand-AtHAB1ΔN ternary complexes (B) Dihedral angles (α = C-C-S-N and β = C-S-N-C) observed for the molecular structure of SB, iSB07 and iSB09 in a small-molecule single crystal (XTAL) and those into the crystals of the complexes HAB1-Ligand-CsPYL1 (WT) and HAB1-Ligand-CsPYL15M (5M).(C) Bar-histograms displaying the a and b torsion angles observed for the structures recorded in the Cambridge Structural Database (CSD). A maximum in the conformer distributions represents the ground energy conformation for a molecule (60, 61). The arrows indicate the corresponding torsion angle as described in (A), black for SB, green for iSB07 and red for iSB09. (D) Molecular structures for the compounds SB, iSB07 and iSB09 showing the displacement ellipsoids for the non-hydrogen atoms drawn at the 50% probability level.

A

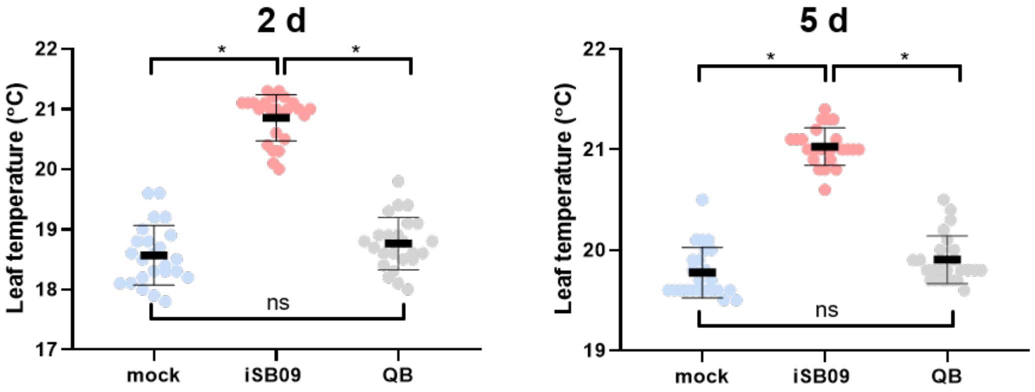

B

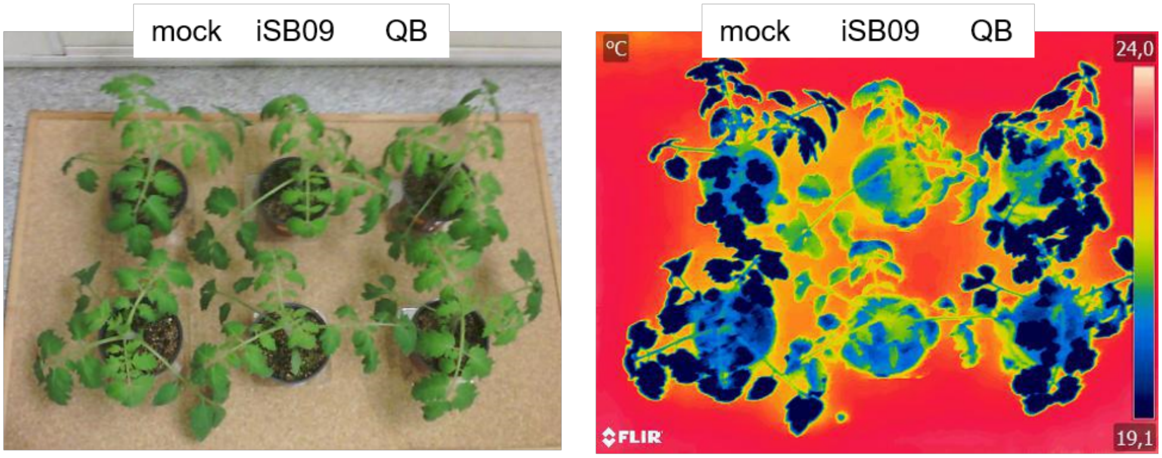

C

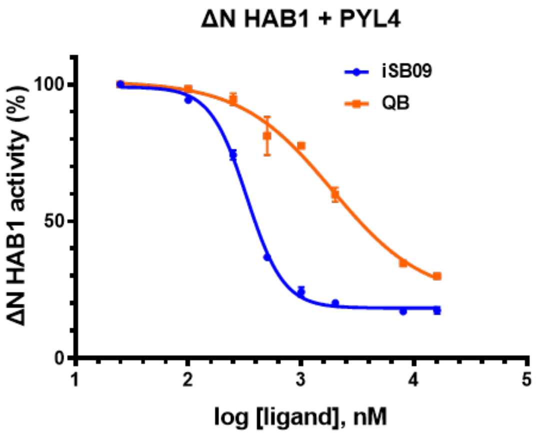

Fig. S6. QB lacks bioactivity in tomato whereas iSB09 is active and shows long-lasting effect.

(A) Quantification of the leaf temperature difference at 2 and 5 days after spraying tomato plants with 0.1 % DMSO (mock-treated control), 10 µM iSB09 or 10 µM QB. Asterisk indicates $p<0.001$ (Student's t test). Values show individual points and average ± SD (n=4 replicates for mock and chemical treatments). (B) IR-images of representative tomato plants at 2 days after being treated with 0.1 % DMSO (mock-treated control), 10 µM iSB09 or 10 µM QB. Photographs were taken with white light (leaf panel) or infrared thermography (right panel) using the camera FLIR E95. (C) iSB09 is more effective than QB in inhibiting HAB1 phosphatase activity in presence of PYL4. IC50 values for iSB09 or QB-dependent inhibition of HAB1 by AtPYL4. Values (average of duplicates) show PP2C activity after incubation with the indicated concentration of ligand and AtPYL4 at 1:2 ratio (1 µM phosphatase:2 µM receptor). The IC50 was 330 nM for iSB09 and 1880 nM for QB. Ligands were assayed at 25, 100, 250, 500, 1000, 2000, 8000 and 16000 nM.

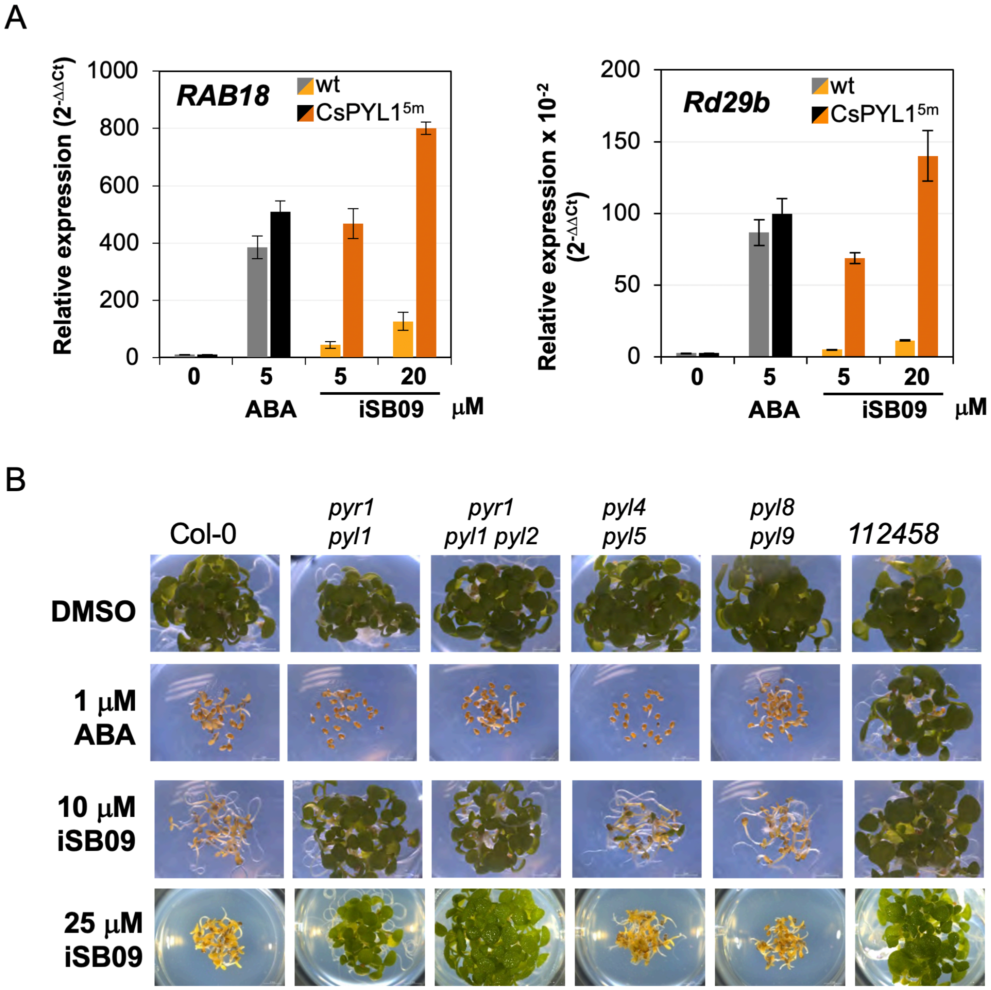

Fig. S7. Enhanced upregulation of *RAB18* and *RD29B* expression by iSB09 in lines overexpressing CsPYL1^{5m}.

(A) RT-qPCR analysis of *RAB18* and *RD29B* expression in response to the indicated concentrations of ABA or iSB09 either in WT plants or lines overexpressing CsPYL1^{5m}. (B) iSB09 requires PYR1 and PYL1 for inhibition of seedling establishment. Effect of iSB09 and ABA either in WT plants or different Arabidopsis mutants lacking the indicated ABA receptors. 112458 is the abbreviation for *pyr1 pyl1 pyl2 pyl4 pyl5 pyl8* sextuple mutant.

**Data Collection**				
Data set **(a)**	**CsPYL1-SB-HAB1**	**CsPYL1^{5M}-SB-HAB1**	**CsPYL1-iSB7-HAB1**	**CsPYL1^{5M}-iSB7-HAB1**
Crystal system, space Group	Orthorhombic, $P2_12_12_1$	Orthorhombic, $P2_12_12_1$	Orthorhombic, $P2_12_12_1$	Orthorhombic, $P2_12_12_1$
Cell dimensions				
A (Å)<br>b (Å)<br>c (Å)	42.96<br>62.73<br>186.93	42.99<br>62.91<br>186.62	43.12<br>62.45<br>187.58	42.82<br>62.72<br>186.95
α,β,γ (º)	90.0	90.0	90.0	90.0
Wavelength (Å)	0.979257	0.979257	0.979257	0.979260
Total refections	115208 (7733)	275264 (26989)	163542 (15137)	171276 (14725)
$^{\#}R_{pim}$ (%)	6.66 (37.82)	2.32 (28.51)	4.63 (38.56)	5.12 (60.28)
$^{+}CC_{1/2}$ (%)	99.2 (78.4)	99.9 (89.5)	99.7 (84.7)	99.5 (69.5)
$\langle I/\sigma(I)\rangle$	7.93 (1.25)	18.89 (2.55)	10.18 (1.57)	7.37 (0.92)
Completeness (%)	98.4 (86.2)	99.7 (99.5)	98.9 (95.0)	98.9 (93.0)
Wilson B-factor	44.67	29.56	35.89	46.85
Multiplicity	5.5 (4.4)	6.1 (6.1)	5.4 (5.3)	5.9 (5.6)
**Refinement**				
Resolution (Å)	44.21-2.37 (2.46-2.37)	44.23-1.84 (1.91-1.84)	44.19-2.1 (2.18-2.1)	44.21-2.13 (2.21-2.13)
Reflections used in refinement	20896 (1773)	44840 (4418)	30200 (2863)	28781 (2636)
R_{work}/R_{free} (%)	18.04 / 24.58 (31.73 / 42.12)	16.80 / 19.87 (25.52 / 29.84)	18.58 / 24.53 (28.90 / 33.72)	19.07 / 24.69 (29.16 / 34.17)
Asymmetric unit content				
Protein residues	490	490	496	488
Ligand/GOL molecules	1 / 3	1 / 1	1 / 3	1 / 4
Manganese/ Chlorine ions	4 / 6	4 / 6	4 / 5	4 / 5
Water molecules	122	250	174	77
Average B factor (Protein / Ligand)	51.5 / 66.2	38.5 / 40.0	51.3 / 52.5	63.0 / 71.6
Rmsd				
Bond lengths (Å) / angles (º)	0.009 / 1.10	0.006 / 0.80	0.008/0.92	0.009/1.03
Ramachandran plot statistics	96.0% in favoured<br>0.0% outliers	97.7% in favoured<br>0.0% outliers	97.1% in favoured<br>0.2% outliers	96.2% in favoured<br>0.2% outliers
**Data Collection**				
Data set **(b)**	**CsPYL1-iSB9-HAB1**	**CsPYL1^{5M}-iSB9-HAB1**	**CsPYL1-QB-HAB1**	**CsPYL1^{5M}-ABA-HAB1**
Crystal system, space Group	Orthorhombic, $P2_12_12_1$	Orthorhombic, $P2_12_12_1$	Orthorhombic, $P2_12_12_1$	Orthorhombic, $P2_12_12_1$
Cell dimensions				
A (Å)<br>b (Å)<br>c (Å)	42.70<br>62.25<br>187.01	42.78<br>62.70<br>187.33	43.34, 62.94, 187.86	42.76, 62.92, 187.01
α,β,γ (º)	90.0, 90.0, 90.0	90.0, 90.0, 90.0	90.0, 90.0, 90.0	90.0, 90.0, 90.0
Wavelength (Å)	0.979257	0.979257	0.9793	0.97926
Total refections	254498 (24492)	310204 (27754)	106878 (10198)	203813 (20268)

$^{\#}R_{pim}$ (%)	4.11 (99.86)	3.44 (61.09)	2.7 (82.06)	5.64 (27.0)
$^{+}CC_{1/2}$ (%)	99.9 (50.2)	99.8 (46.3)	99.9 (54.1)	99.3 (91.3)
$<I/\sigma(I)>$	11.48 (0.90)	12.87 (1.16)	11.69 (0.81)	7.38 (1.49)
Completeness (%)	98.9 (98.3)	99.1 (92.3)	99.6 (96.6)	95.71 (83.74)
Wilson B-factor	35.19	32.04	29.06	45.08
Multiplicity	6.4 (6.3)	6.4 (6.2)	2.0 (2.0)	8.6 (8.6)
**Refinement**				
Resolution (Å)	44.05-1.9 (1.97-1.9)	44.24-1.78 (1.85-1.78)	42.23-1.74 (1.80-1.74)	44.28-2.28 (2.36-2.28)
Reflections used in refinement	39905 (3883)	48565 (4445)	53446 (5108)	22866 (1977)
R_{work}/R_{free} (%)	19.40 / 24.68 (36.27 / 39.84)	17.98 / 21.19 (33.82 / 35.43)	17.72 / 20.33 (33.73 / 37.27)	20.63 / 26.31 (30.06 / 39.02)
Asymmetric unit content				
Protein residues	483	484	487	501
Ligand/GOL molecules	1 / 1	1 / 3	1 / 1	1 / 1
Manganese/Chlorine ions	4 / 6	3 / 6	4 / 3	4 / 6
Water molecules	166	237	271	44
Average B factor (Protein / Ligand)	50.5 / 54.5	42.3 / 50.5	40.7 / 39.1	67.1 / 72.0
Rmsd				
Bond lengths (Å) / angles (º)	0.009 / 0.96	0.008 / 0.94	0.006 / 0.82	0.008 / 1.05
Ramachandran plot statistics	98.1% in favoured 0.2 % outliers	97.5% in favoured 0.0 % outliers	97.5% in favoured 0.0 % outliers	93.9% in favoured 0.6 % outliers

Highest-resolution shell is shown in parentheses.

$$^{\#}R_{p.i.m.} = \sum_{hkl}\sqrt{1/n-1}\sum_{j=1}^{n}\left|I_{hkl}-\langle I_{hkl}\rangle\right| \Big/ \sum_{hkl}\sum_{j} I_{hkl,j}$$

$^{+}CC_{1/2}$ is the correlation coefficient of the mean intensities between two random half-sets of data.

Data set **(c)**	**SB**	**iSB7**	**iSB9**
Chemical formula	$C_{18}H_{19}N_2O_3S$	$C_{18}H_{18}N_2O_3S$	$C_{19}H_{20}N_2O_3S$
M_r	343.41	343.40	356.43
**Data Collection**			
Crystal system	Orthorhombic	Monoclinic	Monoclinic
Space group	$P2_12_12_1$	$P2_1$	$P2_1$
A (Å)	5.233(4)	5.1470(5)	10.696(4)
b (Å)	14.766(5)	15.5400(6)	15.619(4)
c (Å)	20.790(8)	10.2510(9)	10.7320(14)
α, β, γ (°)	90, 90, 90	90, 100.415(7), 90	90, 105.83(2), 90
Volume/Å^3	1606.5(14)	806.41(11)	1724.9(8)
Z , μ (mm^{-1})	4, 0.326	2, 0.324	4, 0.307
Radiation	Synchrotron (λ = 0.82653)		
2Θ range for dat collection(°)	3.934 to 67.882	4.698 to 64.986	4.588 to 65.028
Index ranges	-6 ≤ h ≤ 6<br>-19 ≤ k ≤ 19<br>-26 ≤ l ≤ 26	-6 ≤ h ≤ 6<br>-20 ≤ k ≤ 19<br>-13 ≤ l ≤ 13	-12 ≤ h ≤ 13<br>-20 ≤ k ≤ 19<br>-13 ≤ l ≤ 13

Refl. collected, independent	21743, 3779	9262, 3396	13450, 6688
R_{int} , R_{sigma} (%)	4.47, 2.74	7.94, 8.38	4.15, 5.05
**Refinement**			
Data / restraints / parameters	3779 / 0 / 224	3396 / 1 / 222	6688/1/461
Goodness-of-fit on F^2	1.048	1.115	1.033
Final R indexes [I>=2σ (I)] (%)	R_1 = 3.84, wR_2 = 10.18	R_1 = 5.20, wR_2 = 12.77	R_1 = 5.65, wR_2 = 15.08
Final R indexes [all data] (%)	R_1 = 4.16, wR_2 = 10.46	R_1 = 5.21, wR_2 = 12.78	R_1 = 6.06, wR_2 = 15.59
Largest diff. peak / hole / e $Å^{-3}$	0.31 / -0.48	0.47 / -0.83	0.70/-0.79

Table S1.

Diffraction data collection and refinement statistics for (a) CsPYL1-SB-HAB1, CsPYL1^{5M}-SB-HAB1, CsPYL1-iSB7-HAB1 and CsPYL1^{5M}-iSB7-HAB1, (b) CsPYL1-iSB9-HAB1, CsPYL1^{5M}-iSB9-HAB1, CsPYL1-QB-HAB1 and CsPYL1^{5M}-ABA-HAB1 (output from Phenix generate table) and (c) the ligands, SB, iSB7 and iSB9
